# The impact of the COVID-19 recession on Mexican households: evidence from employment and time use for men, women, and children

**DOI:** 10.1007/s11150-022-09600-2

**Published:** 2022-01-29

**Authors:** Lauren Hoehn-Velasco, Adan Silverio-Murillo, Jose Roberto Balmori de la Miyar, Jacob Penglase

**Affiliations:** 1 Andrew Young School of Policy Studies, Georgia State University, Atlanta, GA USA; 2 School of Government, Tecnologico de Monterrey, Monterrey, Mexico; 3 Business and Economics School, Universidad Anáhuac México, Naucalpan de Juárez, Mexico; 4 San Diego State University, San Diego, CA USA

**Keywords:** COVID-19, Women, Children, Mexico, Labor Supply, Gender, H12, J12, J13, J16, J18, O12

## Abstract

This study examines changes in labor supply, income, and time allocation during the COVID-19 pandemic in Mexico. Using an event-study design, we show that the COVID-19 recession had severe negative consequences for Mexican households. In the first month of the pandemic, employment declined by 17 percentage points. Men recovered their employment faster than women, where men’s employment approaches original levels by 2021Q2. Women, on the other hand, experienced persistent employment losses. Within-household, men also increased their time spent on household chores while neither gender (persistently) increased their time caring for others. Instead, children reduced their time spent on schoolwork by 25%.

## Introduction

The economic consequences of the COVID-19 pandemic have been severe. In addition to death and illness, households have been burdened by shuttered economies, resulting in a dramatic reduction in economic activity. The pandemic has been particularly harmful to economic activity in Mexico, where GDP initially declined by 8.5% and formal employment by 5% (Flores, 2020, IMSS, 2020). Despite the severity of the recession, the Mexican government has offered no new public policies to aid affected groups, unlike counterparts in high-income countries (von Gaudecker et al., 2020) and similar Latin American countries (Hale et al., 2020). Moreover, compared to high-income countries, Mexico has fewer remote work opportunities and weaker public support systems (Peluffo and Viollaz, 2021).

In this paper, we examine the economic consequences of the COVID-19 pandemic in Mexico. Using data from Mexico’s National Employment and Occupation Survey (*Encuesta Nacional de Ocupación y Empleo* or ENOE), we measure changes in employment, income, and time use during the COVID-19 recession. There are several benefits of this data source. First, the ENOE records both formal and informal work, which is essential in a setting such as Mexico, where a significant fraction of the jobs are informal (Alvarez and Ruane, 2019).^1^ Second, in addition to labor supply, the ENOE provides information on household time use, including time spent caring for others and time allocated toward household chores. Third, the ENOE includes measures of school enrollment and time allocation for children. Using children’s time allocation, we can determine how school closures affected schoolwork during the pandemic and observe how households responded to school closures.

We use three primary specifications throughout our analysis. First, we use the traditional ENOE and an event-study design to track the impact of the pandemic and subsequent recovery. Second, because the traditional ENOE transitioned into a telephone survey (the ETOE) during the lockdown period of the pandemic, we use the ETOE to track the same individuals over each month of the pandemic (April through November of 2020) using individual fixed effects. Then, we test the robustness of the main findings using a grouped post-period specification to capture the average effect over the course of the pandemic recession. In all three specifications and across all individuals, the COVID-19 pandemic reduced employment and income, with little impact on adult household time, except for an increase in men’s time spent on household chores.

In the traditional ENOE, employment at both the extensive and intensive margins declines initially but almost fully recovers by the end of 2021Q2 for men. Women’s labor supply is still below initial levels by the end of our data series. The majority of employment gains occur in the informal sector. The results with the ETOE largely reflect these findings from the traditional ENOE. However, the initial impact of the pandemic is more substantial in the ETOE, likely because it covers the lockdown phase of the pandemic. The results using the ETOE show that employment dropped by 17 percentage points in the first month of the pandemic (April), and hours spent working declined by 13 h per week.

We also estimate changes in household time allocation. In both the traditional ENOE and the ETOE, men reallocate their time to household chores, but not toward time spent caring for others. On average, women do not change their time use across household chores or caring for others (including children). However, women’s time allocated toward household chores seems to be on a downward trend prior to the pandemic, making the impact of the pandemic on this activity challenging to disentangle from the preexisting trend. We also find a slight increase in mothers’ caring time (for those with school-aged children) in 2020Q4. However, the effect only occurs temporarily rather than persistently over the entire pandemic. We attribute the smaller than expected change in household work to the fact that mothers with young children were already spending 45 h per week on household chores and caring for children prior to the pandemic, while mothers with children under 15 were already allocating 38 h per week. Thus, there is little margin for mothers with children to increase their time in household work.

To further investigate these conclusions, we explore the within-household burden on women. We examine the relative contributions to income, hours worked, and household chores across men and women within the same household. We focus on households with a husband, a wife, and children present to capture the impact on household time allocation. In past recessions, women have tended to compensate for their husbands’ employment loss by increasing their own labor supply. Given the uniqueness of the COVID-19 pandemic recession, this insurance mechanism may not apply. Our results demonstrate that the relative contribution of women’s income and labor supply is similar before and after the pandemic. For measures of within-household time use, men contribute more to household chores than before the pandemic. Still, there is no shift in time spent caring for others (including children).

We then conclude by considering the effects of the COVID-19 pandemic on children. Our analysis of adult time use demonstrates that neither men nor women shift their time toward childcare. The lack of change in adults’ time caring for others suggests that children may have responded to school closures by spending less time on schoolwork. To test how children’s time use changed after the pandemic, we select a sample of individuals who are ages 6–16. During the pandemic, children spent eight hours less per week on schoolwork, a reduction of more than 25% from the pre-pandemic mean. However, because Mexico’s public education transitioned to television and online learning (for certain schools) during the pandemic, children may have shifted their time use from school to educational programming (Córdoba and Montes, 2020, Rivers and Gallón, 2020). Unfortunately, our data do not allow us to decompose educational activities into time in school versus television and online learning.

The remainder of this paper is organized as follows. Section 2 reviews the existing literature on the relationship between the COVID-19 recession and labor markets. Section 3 discusses the Mexican context. Section 4 describes the household survey data used throughout the analysis. Section 5 outlines the empirical strategy. Section 6 presents the main results from an event-study specification. Section 7 presents robustness checks. Section 8 presents additional findings within the household and for children. Section 9 concludes.

## Related literature

This study contributes to several areas of current work. First, our study relates to recent work documenting the pandemic’s effect on women, especially mothers. Second, our paper adds to the body of work documenting the economic consequences of the COVID-19 pandemic in a middle-income setting. Third, our research presents evidence on the effects of the COVID-19 pandemic on children.

Unlike previous recessions, the COVID-19 pandemic has been uniquely devastating to women (Albanesi and Kim, 2021, Alon et al., 2020a, b, Croda and Grossbard, 2021, Pitts, 2021). Part of the disproportionate burden on women is attributable to the impact of the pandemic on the service sector, which typically employs more women than men (Alon et al., 2020b). Another portion of the female-focused burden is due to school closures and childcare responsibilities (Alon et al., 2020b, Croda and Grossbard, 2021, Heggeness, 2020, Yamamura and Tsustsui, 2021).^2^ For example, Heggeness (2020) shows that U.S. mothers in early-pandemic closure states experienced a much larger decline in employment relative to other individuals. Similarly, in Germany, mothers’ mental health and well-being declined during the pandemic (Czymara et al., 2020, Mathias, Sevrin, Spiess, Nico A and Gert G, 2021). The mental health decline for mothers is largely attributable to mothers bearing the disproportionate brunt of childcare responsibilities.^3^

Still, much of the above narrative has focused on high-income countries, while middle and low-income countries face different constraints, have more limited opportunities for remote work (Dingel and Neiman, 2020, Peluffo and Viollaz, 2021), and may have less generous programs designed to alleviate the pandemic’s economic harm (such as income support Hale et al. (2020)). Of particular relevance for our study is the feasibility of working from home in middle-income settings. A growing body of work has demonstrated differences in the ability to work from home between developed and developing settings (Bana et al., 2020, Duman, 2020, Charles, Grobovšek, Poschke and Saltiel, 2021, Syed M, Rehman and Zhang, 2021, Mongey and Weinberg, 2020, Peluffo and Viollaz, 2021, Saltiel, 2020, Garrote Sanchez et al., 2021). For example, Saltiel (2020) shows that only 13% of workers in developing countries can work from home. Similarly, Gottlieb et al. (2021) makes the case that only 20% of urban workers can work from home in developing countries versus 40% in wealthy settings. The possibility of working from home is also likely to be correlated within the household (Peluffo and Viollaz, 2021). Peluffo and Viollaz (2021) focuses specifically on Mexico and demonstrates that two partner households have high within household correlations of working from home, which may contribute to higher between-household inequality during the pandemic.

More generally, our study adds to the literature studying the impacts of the COVID-19 pandemic on labor markets in middle-income settings. Existing work in this area has documented both the aggregate impacts and household-specific effects of the pandemic recession (Afridi et al., 2021, Barker et al., 2020, Bhatt et al., 2020, Campos-Vazquez et al., 2020, Estupinan et al., 2020, Khamis et al., 2021, Kugler et al., 2021, Levy and Menezes Filho, 2021, Mohapatra, 2020, Schotte et al., 2021, Silverio-Murillo et al., 2020). One notable study, Kugler et al. (2021), investigates 40 countries and finds the largest employment losses for younger workers, female workers, and those with lower levels of education. Kugler et al. (2021) also documents a labor market recovery between April and August of 2020. Important for our setting, Kugler et al. (2021) emphasizes the importance of real-time phone surveys (despite the lack of representativeness) for understanding the immediate impacts of the pandemic.

Finally, this paper contributes to the literature studying the time spent by children on schooling during the COVID-19 pandemic. A report from the United States Department of Education states that 85% of all school districts expected instructional time to fall under 4 h, which accounts for 1 h per day less than pre-pandemic levels (USDE, 2021). Bansak and Starr (2021) finds that time spent by parents schooling their kids in the United States was actually higher for parents with a high school degree than parents with a college degree. Evidence from school closures in Germany points to a decrease from 7.4 to 3.6 h per day (Grewenig et al., 2020). In contrast to the United States, less educated parents in Germany spend less time schooling their children (Dietrich et al., 2021). Finally, worldwide data points to a loss between 0.3 and 1.1 years of schooling due to school closures during the COVID-19 pandemic (João Pedro, Hasan, Goldemberg, Geven and Iqbal, 2021).

## The Mexican context

### Timeline and public policies during the COVID-19 pandemic

**Fig. 1:**
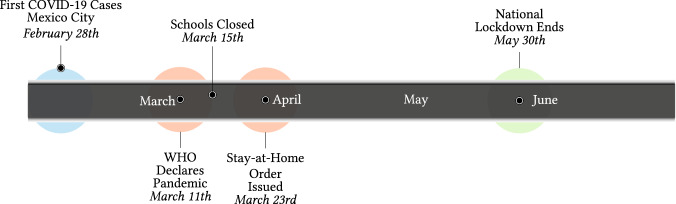
Timeline of Mexico’s Initial Pandemic and National Lockdown

In Mexico, the COVID-19 pandemic began in March of 2020. At this time, schools closed, mobility dropped, and the national stay-at-home order was issued. This national lockdown was then subsequently lifted on May 30th of 2020. To illustrate the sequence of events, we outline the timeline of the pandemic in Fig. 1.

During the lockdown and recovery phase, the Mexican government did not introduce new safety nets. This contrasts with the majority of high-income and Latin American countries (Hale et al., 2020). For instance, among Latin American countries, Peru and Uruguay were quick to pass income support legislation, with Argentina, Bolivia, and Paraguay following. Then by April, Ecuador, Chile, Honduras, Colombia, and Guatemala had also passed income support policies.

Instead, the Mexican government introduced two mitigation policies, neither of which involved any monetary transfer (Lustig et al., 2020). First, individuals could receive an advanced 2-month payment from the non-contributory pension system. Second, credits were given to small and medium-sized enterprises in the formal and informal sectors. For the formal sector, these credits were capped at 25,000 MXN (1100 USD) in total, with a maturity of 3 years, at a 6.5% annual interest rate. México Evalúa (2020) suggests that the advanced payment is equivalent to 0.2% of GDP and the credits to 0.1% of GDP.

Outside of direct support to households and businesses, the Central Bank (Banco de México) also took action to mitigate economic exposure. The Central Bank added bond swaps and loosened rules for minimum deposits among commercial banks. Campos-Vazquez et al. (2020) suggests that the Central Bank’s policies provided liquidity equal to 3.3% of GDP. In addition, the Central Bank granted regulatory flexibility to commercial banks, which allowed banks to give payment extensions to their customers on mortgages, credit cards and commercial loans, waving interest rates and fees for 4 months, beginning in April of 2020. Even with such measures, Mexico’s response has been fairly limited in comparison to other nations. This inability to act left households without government support during a turbulent time. As a result, individuals may have been less willing to leave the labor force during the pandemic relative to similarly-situated countries.

### Unique features of Mexico’s labor markets

The Mexican labor market differs from the labor markets in other countries in several important ways. First, employment in Mexico is heavily concentrated in the informal sector relative to salaried work in the formal sector (Busso et al., 2012, Levy, 2010). Mexican laws regulating salaried and non-salaried workers are often constraining, and this regulation may push jobs into the informal sector (Alvarez and Ruane, 2019). In the formal sector, workers have protections against firings, such as stiff penalties for firms laying off workers (Levy, 2010). While informal work is not directly illegal, workers employed by informal firms are not technically employees (Levy, 2010). Due to the lack of employee status, informal firms are not subject to the same legislation. For example, informal firms do not have to pay the minimum wage, workers cannot organize into unions, and firms do not contribute to social security (Busso et al., 2012, Levy, 2010). Adjustment costs are therefore lower in the informal sector, and we may expect a greater decline in employment for those workers. Due to the relative size of the informal sector in Mexico relative to high-income countries, we may observe different patterns across contexts. To that point, since women have historically had higher participation in informal and unpaid work than men (Ortega-Díaz, 2020), the informal economy is an especially important factor in the context of the gendered recession.

Second, women in Mexico have lower labor force participation than in high-income countries. Historically, women in middle and low-income countries have had lower labor supply (Goldin, 1994), which is at least partially due to cultural norms against women working, especially in Latin America (Arceo-Gomez and Campos-Vazquez, 2010). Today, just under 50% of women participate in the labor force in Mexico (Bustelo et al., 2019, Novta and Wong, 2017), with higher labor supply from single, younger, and more educated women (Bustelo et al., 2019, Hoehn-Velasco and Penglase, 2021, Novta and Wong, 2017). Married women have the lowest labor force participation (Psacharopoulos and Tzannatos, 1993), at <45% in the ENOE survey over 2007–2019 (Hoehn-Velasco and Penglase, 2021). The low labor supply of married women suggests that women may not face the same trade-offs as in high-income countries when schools and childcare centers closed during the pandemic.

Third, women (and especially low-income women) face barriers to childcare access in Mexico. Previous work has demonstrated that access to childcare can be alleviated with public programs (Ángeles et al., 2011, Calderon, 2014, Mateo Díaz and Rodriguez Chamussy, 2013). When women gain access to childcare, they increase their labor supply. Thus, if childcare access increases female labor supply, we might expect the opposite effect when childcare centers close during the pandemic. This lack of childcare access should have similar consequences to high-income countries during the pandemic and potentially pressure women out of the workforce.

Finally, another factor that may increase women’s employment during the COVID-19 pandemic is the added worker effect. In middle and low-income countries without unemployment insurance, women may be faced with economic pressure to enter the labor force when their husband becomes unemployed (Kohara, 2010, Novta and Wong, 2017). This pressure may push women into the labor force during downturns (relative to men) (Novta and Wong, 2017, Skoufias and Parker, 2006). Previous work has shown that women’s labor supply may increase during a recession. For example, Skoufias and Parker (2006) demonstrated that during Mexico’s peso crisis wives, increased their labor supply to compensate for their husbands’ job loss.

### Composition of the Mexican economy

Due to the importance of the service sector in the COVID-19 recession (Alon et al., 2020b), it is important to note the compositional differences between the Mexican economy and that of high-income countries. Mexico’s economy is less reliant on the service sector relative to other more developed nations. According to the CIA factbook, Mexico’s economy is only 64.5% service sector, while the United States is 80% service sector (Agency, 2017). Industry makes up 31.9% of the economy in Mexico, whereas it makes up 19.1% of the economy in the United States. Agriculture makes up the remaining portion of the economy, 3.6% in Mexico and 0.9% in the United States. The importance of person-to-person contact and women’s role in service jobs makes the differences in the composition of the economy between the United States and Mexico relevant for interpreting the findings of this study. Due to the structural differences in the economies, and the lower reliance on the service sector, women’s labor supply adjustments in Mexico may be distinct from high-income countries.

### Schooling in Mexico during the COVID-19 pandemic

School closures in Mexico due to the COVID-19 pandemic began on March 23rd, 2020. It was not until May 2021, with the roll-out of vaccination among teachers, that schools in Mexico opened again.^4^ During the pandemic, school closures in Latin America have been the highest in the world, with an average duration of 217 days. In comparison, the length of closures were more limited in the Middle East and North Africa (167 days), Sub-Saharan Africa (116 days), Asia (107 days), Europe (93 days), and the U.S. and Canada (46 days) (de Hoyos, 2021).^5^ Prior to the re-opening of schools in May 2021, Mexico ranked eighth among countries with the most days of school closures (UNICEF et al., 2021).

To address the school closures, the Mexican government implemented televised learning activities (*aprende en casa*), which is a unique feature of the Mexican context. The government complemented this type of instruction with online activities. However, survey data measuring the impact of the pandemic on education in Mexico from the National Statistics Office (INEGI), known as ECOVID-ED, indicates that most households chose online learning over televised learning. Moreover, many households purchased equipment specifically for distance learning, such as smartphones, laptops, televisions, and tablets.^6^ While we cannot make comparisons to pre-pandemic purchase levels, the fact that technology purchases were common, highlights the shift in education modalities that occurred as a result of school closures.

In terms of enrollment, ECOVID-ED indicates a small drop, particularly in primary school (−1%) and middle school (−5%), while larger reductions occurred in high school (−12%) and college (−7%). Students that dropped out declared distance learning as the primary reason (26.6%), followed by economic hardship due to a parent’s unemployment (25.3%), not having access to the internet or an electronic device (21.9%), definite school closure (19.3%), COVID-19 illness (4.9%), or not having a tutor (4.4%). Regarding public versus private schooling, around 90% of students enrolled in public schools, while the remaining 10% stayed in private institutions throughout the pandemic.

Finally, there is evidence that parents assisted their children with schooling activities and the degree to which this occurred varied by child age. For preschool students, 98.7% had help from a household member. Similarly, 93% of primary school students had one of their parents assisting them, while 51.7% of middle school students had educational support from their parents. Mothers of young children were more involved in school activities compared to mothers of older children, with the share of maternal involvement being 84.0%, 77.0%, and 60.2% for preschool, primary school, and secondary school, respectively. Interestingly, fathers tended to be more involved in the education of older children. The percentage of fathers supporting education at each level was 5.9% for preschool, 7.9% for primary school, and 10.2% for secondary school. Again, we cannot compare these figures to their pre-pandemic levels, but they demonstrate the potential importance of school closures on labor supply.^7^

## Data

### ENOE data description

We use quarterly data from Mexico’s National Employment and Occupation Survey (*Encuesta Nacional de Ocupación y Empleo* or ENOE). This data is available from 2005 and onwards. The ENOE follows individuals in a rotating panel. Each wave, one-fifth of households move into and out of the survey. The ENOE tracks household composition and the characteristics of each member in the household. The survey records each members’ education, labor force participation (hours worked and employment), time use on several household activities, monthly income, and key demographic characteristics.

During the COVID-19 pandemic, data collection of the ENOE turned into a telephone survey. For the 3 months of April, May, and June, the ENOE became the Telephone Survey of Occupation and Employment (*Encuesta Telefónica de Ocupación y Empleo* or ETOE). This survey occurred monthly rather than quarterly, with the same households followed over each month.

At the start of quarter three (July 2020), the ENOE-N began. The ENOE-N collects both the traditional quarterly ENOE and the monthly telephone version of the survey in the ETOE. Thus, beginning in July 2020 (quarter three), we have each:

1. The monthly ETOE data with the same individuals followed from April to November 2020.^8^ Note that the ETOE follows the same households and is not a rotating scheme.
2. The traditional quarterly ENOE from 2020Q3 to 2021Q2. The traditional ENOE starting in 2020Q3 resumes face-to-face format and the rotating scheme.

The ETOE and the ENOE survey methods are each provided in the ENOE-N. However, the ENOE-N distinguishes interview types from each other. The quarterly face-to-face ENOE corresponds to type 1 in the ENOE-N, and the monthly telephone ETOE corresponds to type 2 in the ENOE-N.

In our main analysis, we focus on the traditional face-to-face interviews in the ENOE. The ENOE may be more representative of Mexico as a whole as the survey methods rely on face-to-face interviews. By comparison, the ETOE relies on telephone interviews and requires households to have listed a telephone number on the survey. Thus, our main sample includes the traditional ENOE for 2019Q1 through 2021Q2, without any information for 2020Q2. A limitation of this chosen approach is that the information in the traditional ENOE includes no information about the immediate impacts of the COVID-19 pandemic. Because the lockdown period of the pandemic occurred only in 2020Q2, our main results will not contain information on household labor supply or time use during the lockdown phase.

To overcome this limitation, we use the ETOE (in addition to the ENOE) to study the immediate effects of the pandemic, following the same individuals over time. The main limitation of the ETOE sample is that it will only represent individuals who have access to a telephone. Still, the ENOE maintains that the ETOE is representative at the national level. However, the ENOE does warn of potential bias in the ETOE for particular subsamples and outcomes. For this reason, we only consider subsamples of adult men and women for the main outcomes of labor supply and time use, and we avoid breaking the sample into smaller subsamples (e.g., households with children). Observable differences between the ETOE and the ENOE are illustrated in Table B.1 and Table B.2. In both tables, individuals in the ETOE are slightly older and less likely to have a less than primary education.

While the ETOE follows the same individuals over time, and we implement individual fixed effects in the analysis, another limitation of the ETOE data is that individuals leave the sample for various reasons. We test whether individual demographic characteristics predict the probability of attrition from the ETOE in Table B.3. Individual characteristics do not appear to predict attrition from the ETOE by the end of the survey period.

### Summary statistics

Table 1 presents descriptive statistics for our analysis sample from the traditional ENOE. We split the sample into men and women over the pre and post-period of the COVID-19 pandemic. The summary statistics shown represent 2019Q1–2020Q1 in the pre-pandemic period and 2020Q3–2021Q2 in the post-pandemic period. The sample includes individuals ages 18–64, a total sample of two million observations.

**Table 1:** Descriptive statistics traditional face-to-face ENOE, adults 18–64

	Women Pre		Women Post		Men Pre		Men Post	
	Mean	St. Dev.	Mean	St. Dev.	Mean	St. Dev.	Mean	St. Dev.
Employment								
1(Working)	0.500	0.500	0.469	0.499	0.835	0.371	0.807	0.395
Hours Worked	18.526	22.379	17.066	22.165	37.645	22.925	35.356	23.552
1(Unemployed)	0.019	0.135	0.022	0.146	0.031	0.173	0.039	0.193
Sector								
1(Formal)	0.223	0.416	0.215	0.411	0.391	0.488	0.371	0.483
1(Informal)	0.277	0.448	0.254	0.435	0.443	0.497	0.436	0.496
1(Construction/Manufac.)	0.084	0.277	0.082	0.274	0.254	0.435	0.249	0.433
1(Trade)	0.125	0.331	0.121	0.326	0.126	0.332	0.122	0.327
1(Service)	0.270	0.444	0.245	0.430	0.307	0.461	0.289	0.453
1(Agriculture)	0.017	0.129	0.018	0.133	0.133	0.340	0.133	0.340
1(Other)	0.004	0.063	0.003	0.057	0.014	0.118	0.014	0.115
Time Use								
Hours on House	24.456	13.320	23.121	13.298	7.352	6.322	7.690	6.917
Hours on Chores	20.933	11.985	20.507	12.484	4.928	5.219	5.278	5.723
Hours on House Maintenance	0.033	0.614	0.054	0.804	0.552	1.994	0.685	2.454
Hours Home Purchasing	2.741	2.749	2.434	2.825	1.543	2.008	1.563	2.165
Hours on Building	0.004	0.344	0.006	0.415	0.059	1.109	0.071	1.215
Hours Caring for Others	7.416	12.648	7.315	13.215	2.081	5.627	2.142	6.185
Hours on Schoolwork	2.481	9.619	2.153	8.395	3.464	11.289	2.826	9.473
Hours Transporting	0.746	2.001	0.120	1.067	0.270	1.217	0.093	0.791
Hours on Community	0.071	1.079	0.055	1.032	0.100	1.405	0.087	1.374
Income								
Monthly Income	1923.4	3993.9	1998.2	4400.1	4189.9	6044.7	4294.8	6502.5
Characteristics								
Age	38.668	13.125	38.840	13.195	38.055	13.215	38.211	13.259
1(Education-Less than Primary)	0.037	0.188	0.036	0.185	0.029	0.167	0.028	0.166
1(Education-Primary)	0.206	0.404	0.198	0.399	0.195	0.397	0.190	0.392
1(Education-Middle)	0.284	0.451	0.281	0.450	0.284	0.451	0.279	0.449
1(Education-High School)	0.213	0.410	0.217	0.412	0.245	0.430	0.244	0.430
1(Education-Above High School)	0.260	0.439	0.269	0.443	0.247	0.431	0.259	0.438
N	646,690		403,611		584,955		363,076	

The sample includes men and women in the traditional ENOE data who are 18–64

Individual-level data from the traditional face-to-face National Occupation and Employment Survey (ENOE) 2019Q1–2020Q1 and 2020Q3–2021Q2

Prior to the pandemic, roughly 50% of women were employed. Then, following the onset of the pandemic, the share of employed women falls to 46.9%. For men, 83.5% were employed before the pandemic and 80.7% after. On the intensive margin of employment, hours worked per week declines by 1.5 h for women and 2 h for men. This reduction in hours worked (per week) reflects both lower hours for employed workers and extensive margin job loss.

We also consider time-use categories collected by the ENOE. We define time-use categories based on the ENOE definitions, where individuals report both hours and minutes spent on each category of time use.^9^ The eight categories include time spent on chores, household purchasing/accounting, transporting members of the family, building the home, schoolwork, maintaining the home, community service, and finally caring for others (including children).^10^ We present the exact question asked for each of the eight categories in Appendix Fig. A.1. For adults, we focus on time spent caring for others and time on household chores. As a robustness check for households with children, we use the more detailed categories.

Table 1 shows that women slightly decreased their time spent on general household production during the pandemic (by 1 h). Women similarly marginally decreased their time spent caring for others (children, the sick, the elderly) and on household chores. Men, on the other hand, increased their time caring for others and on household chores. For the remaining categories of time use, women report less time on almost every activity. By comparison, men increased their time on nearly every activity except transportation and community service.

**Fig. 2:**
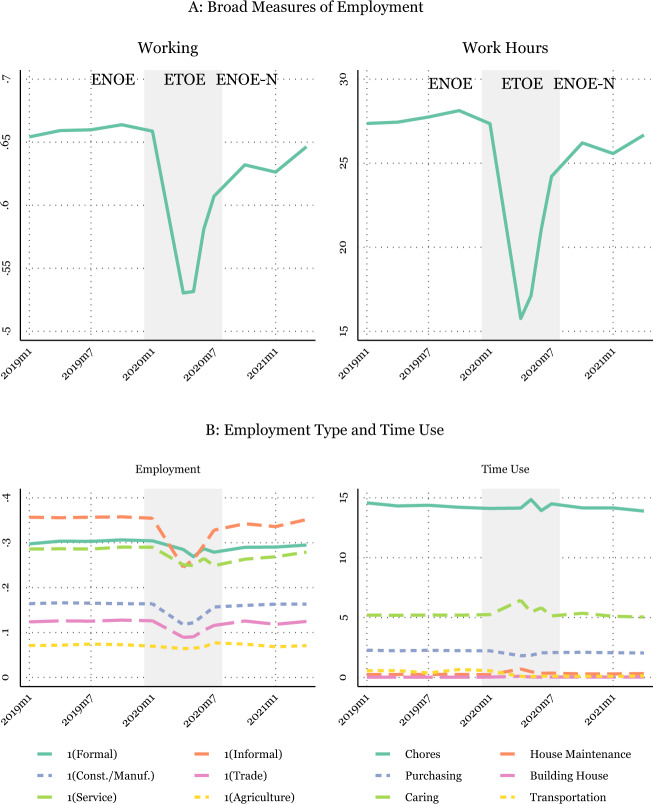
Time Series of Labor Supply and Time Use, ENOE and ETOE. NOTES: Individual-level data from the traditional face-to-face National Occupation and Employment Survey (ENOE) 2019Q1–2020Q1 and 2020Q3–2021Q2. Shaded gray area shows the months added from the ETOE. Sample weights are applied when collapsing the data based on the ENOE’s specified sample weights

We also plot the primary measures of employment and hours worked overtime (on average), combining the ETOE and ENOE in Fig. 2. The plotted time series shows the ENOE in the white area of the graph, with the immediate effects of the pandemic shown by the ETOE in the gray shaded portion of the chart. Based on the information provided, the ETOE captures the worst months of the pandemic, including the initial lockdown period. Figure 2 illustrates the limitation of only relying on the ENOE, which will solely capture the effect of the pandemic after the second quarter of 2020.

### Other subsamples

In addition to the main sample of adults 18–64, shown in the above summary statistics, we also perform several subsample analyses. First, of particular interest is the ETOE sample, which captures the lockdown phase of the pandemic. We present the summary statistics for the ETOE in Table B.4, over adults age 18–64. The ETOE shows that the immediate impact of the pandemic was more severe, with a substantial reduction in employment for both men and women.

Second, while our paper focuses on labor supply and time use for adults, we also study the effects on children age 6–16 in additional results. Table B.5 shows the descriptive statistics for children age 6–16. During the pandemic, children show a large drop in time spent on schoolwork (8 h). Despite the change in school time, there is a lower relative change in reported school attendance. Boys also show an increase in the probability of working and hours worked in the pandemic period.

Third, in the main results, we present the results for mothers and fathers by child age. To ensure that we have correctly mapped parents to children, for analyses labeled as mothers and fathers, we only include household heads, their spouses, and the direct children of the household head. We take the subsample of household heads and their spouses because this ensures we correctly link children to their parents. Further, in additional results, we restrict our sample to nuclear families which are defined as households that include a household head, their spouse, and the associated children of the household head (i.e., no extended family members).

## Main empirical strategy

### Event-study specification

We primarily rely on an event study to investigate the impact of the pandemic on household time use and labor supply. Considering quarterly changes in each outcome using an event-study design allows us to observe adjustments over each survey wave, capturing any recovery in the labor market from July 2020 to the second quarter of 2021. As opposed to a difference-in-differences specification, which yields the average effect over the post-period, the event-study design displays the time-varying impact of the pandemic.

More formally, our event-study specification appears as:

1 $${Y}_{ist}={\alpha }_{s}+\mathop{\sum }\limits_{Q=-5}^{3}{\beta }_{Q}\,{{{\mbox{COVID}}}}_{Q}+{{{\bf{X}}}}^{\prime} \gamma +{\epsilon }_{ist}$$

where *Y*_*ist*_ is the outcome of interest and includes labor supply, income, and time use for individual *i* in state *s* during quarter *t*. The main effect of the pandemic recession is captured by the event-study indicator variable, COVID_*Q*_. *Q* represents the period relative to the start of the pandemic, and covers five quarters before (2019Q1–2020Q1) and four survey waves (quarters) after the start of the pandemic (2020Q3–2021Q2). We exclude the quarter before the onset of the COVID-19 pandemic, *Q* = −1, as the baseline period, which represents 2020Q1. Each plotted point before and after the excluded period is relative to 2020Q1.

We also include individual-level controls, *X*_*it*_. Controls include indicators for the individual’s age and indicators for the individual’s education level.^11^*α*_*s*_ captures the state-level fixed effects. *ϵ*_*ist*_ is the standard error, which we cluster at the state level. We do not include time fixed effects in the above specification, as there is no variation in timing within each event-study indicator.

## Results

### Event-study impacts on men and women

**Fig. 3:**
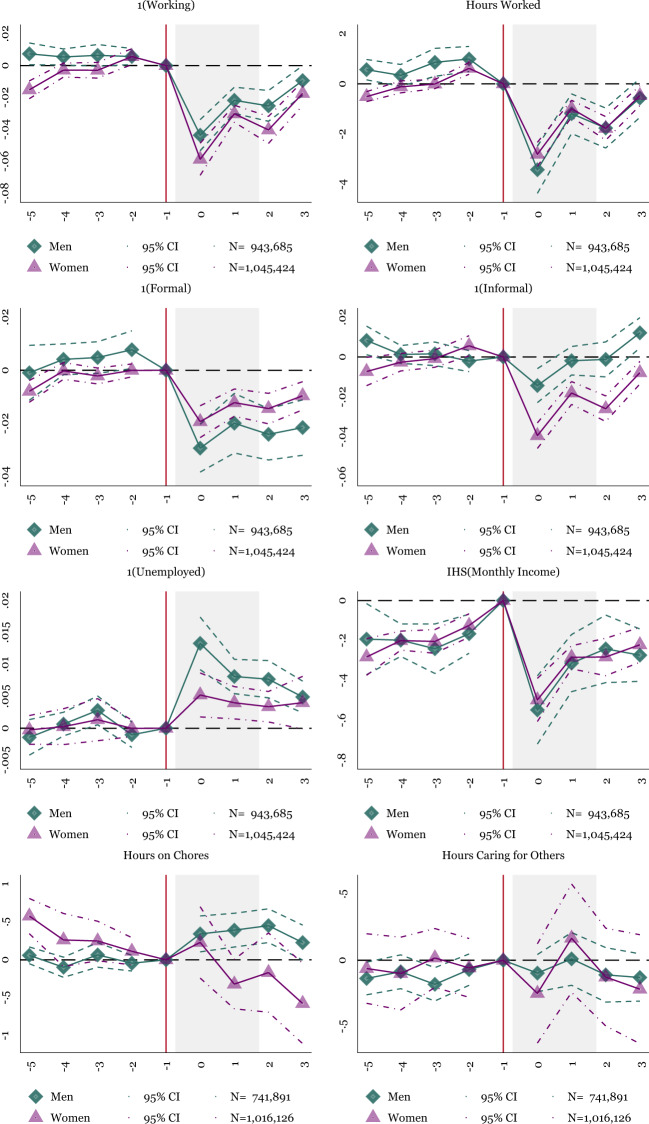
Event Study: Labor Supply, Income and Time Use for Men and Women, 18–64. NOTES: OLS coefficients reported from Eq. (1). Baseline fixed effects include state-level fixed effects. Controls include indicators for the individual’s age and education. Sample weights are applied using the ENOE’s specified sample weights. The periods before −1 include 2019Q1–2019Q4. The omitted period represents 2020Q1, represented by the vertical line. There is no 2020Q2, so the post periods represent 2020Q3–2021Q2. The shaded period represents quarters in 2020. The main sample includes individuals who are 18–64. Robust standard errors are clustered at the state level. Individual-level data from the traditional face-to-face National Occupation and Employment Survey (ENOE) 2019Q1–2020Q1 and 2020Q3–2021Q2

**Fig. 4:**
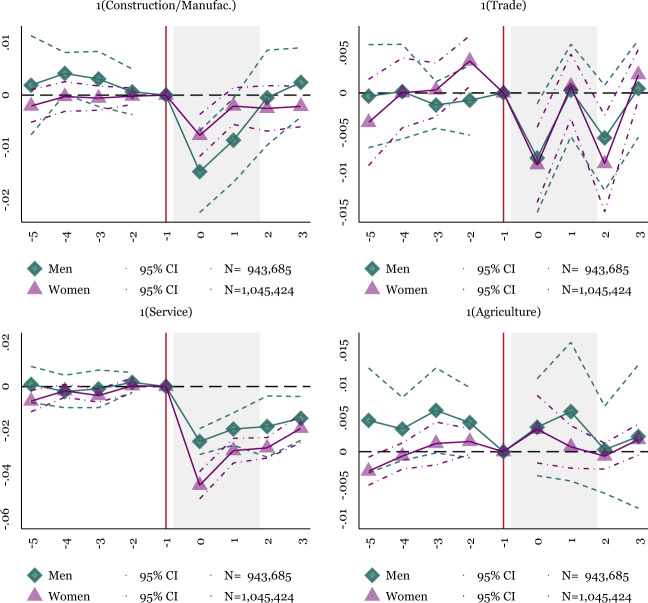
Event Study: Employment Losses by Sector for Men and Women, 18–64. NOTES: OLS coefficients reported from Eq. (1). Baseline fixed effects include state-level fixed effects. Controls include indicators for the individual’s age and education. Sample weights are applied using the ENOE’s specified sample weights. The periods before −1 include 2019Q1–2019Q4. The omitted period represents 2020Q1, represented by the vertical line. There is no 2020Q2, so the post periods represent 2020Q3–2021Q2. The shaded period represents quarters in 2020. The main sample includes individuals who are 18–64. Robust standard errors are clustered at the state level. Individual-level data from the traditional face-to-face National Occupation and Employment Survey (ENOE) 2019Q1–2020Q1 and 2020Q3–2021Q2

Figures 3 and 4 present the main event-study results from Eq. (1). The sample includes all adult men and women (separately) from the ENOE who are 18–64. The green diamond plotted points reflect men, and the purple triangle plotted points show the impact on women. The gray shaded area represents survey waves within 2020 (potentially the worst months of the pandemic). The excluded reference period, 2020Q1, is represented by the vertical line.

Figure 3 displays the main impact of the COVID-19 pandemic on labor supply and time use. Focusing on the labor supply consequences of the pandemic (at the top of Fig. 3), both men and women experience substantial negative impacts on their labor supply. Employment falls by roughly five percentage points in the first (available) quarter (2020Q3), with women experiencing more considerable employment losses than men. Men also recover their employment slightly faster than women and are almost back to baseline levels by 2021Q2. Despite the more evident impact on women’s extensive probability of employment, men’s and women’s hours worked decline (and recover) similarly. Hours spent working are nearly back to baseline level by 2021Q2 for both men and women.

Because Mexico has a robust informal sector, and Mexican women are more likely to be participating in informal and unpaid work (Ortega-Díaz, 2020), we separate the impact on employment by the informal and formal sectors. The results in the second two panels of Fig. 3 show a more apparent and persistent impact on formal employment than informal employment. Formal employment declines by 2–3.5 percentage points and fails to recover by 2021Q2. Instead, workers appear to shift into informal work, where both men’s and women’s employment recovers relatively quickly. By the end of 2020, men have entirely recovered their initial levels of informal employment, and by quarter two of 2021, they have surpassed the initial levels of informal employment. These graphs suggest that informal employment leads Mexico’s labor market recovery. Despite the benefits of the quick employment gains in the informal sector, the loss of formal sector jobs has potential costs to workers. Informal laborers have few employment protections; there is no minimum wage, access to unions is nonexistent, and there are few protections over firings (Busso et al., 2012, Levy, 2010). Thus, while labor markets have recovered, the majority of the gains are in positions with few guarantees and protections for workers.

We then present measures of unemployment in addition to measures of employment. While there are clear job losses during the pandemic, some workers may choose to leave the labor market entirely. Parents may exit the labor force to care for children, and other workers may decide that the risk of infection is too high to justify a job search. The fifth graph of Fig. 3 shows that unemployment rises for both men and women, but slightly more for men. For both groups, the rise in unemployment is at most 1.5 percentage points, less than the employment loss of 5 percentage points. The unemployment results indicate that some individuals may exit the labor force instead of searching for a new position.

To better understand the job loss by the economic sector, Fig. 4 breaks out employment into sectors, including service, construction and manufacturing, trade, and agriculture. The service sector shows the most considerable employment reductions for men and women, with greater employment losses for women. Construction and manufacturing employment also dip in the third quarter of 2020 but begin to recover by the end of 2020, with the recovery more apparent for men. Trade and agricultural sectors show less evidence of employment declines. The importance of employment losses in the service sector reflects similar findings in the United States (Alon et al., 2020b). Our results suggest that the pandemic’s impact on the service sector is important for both men’s and women’s employment, with women more affected than men. While the service sector in Mexico is smaller than the United States (64.5% versus 80%, Agency (2017)) service jobs still show significant reductions by 3–4 percentage points.

Then, moving back to Fig. 3, we consider the impact of the pandemic on individual income. We take the inverse hyperbolic sine of income to approximate a natural log distribution while maintaining zero earners (Bellemare and Wichman, 2020). Income declines substantially in the first available quarter (for both men and women) but almost wholly recovers by the end of the data series. The pattern of income loss is similar across both men and women.

Then, in the bottom two panels of Fig. 3, we show measures of time use. Men exhibit the clearest adjustment as they spend more time on household chores after the pandemic begins. Despite the increase in time spent on chores, men allocate no extra time toward caring for others (including children). For women, there is no significant reallocation of time toward household chores or time toward caring for others. Though time spent on household chores appears to be on a secular decline before the start of the pandemic, making the precise impact of the pandemic difficult to isolate.

Overall, the findings suggest that both men and women experienced considerable reductions in employment, hours worked, and income, but men recover their employment faster, especially in the informal sector of the economy. The only puzzling fact appears in Fig. 3, where women fail to reallocate their time to household chores or toward caring for others. However, due to the preexisting decline in time on household chores, the actual effect of the COVID-19 pandemic is difficult to disentangle. We next explore whether this response differs for households with children.

### Impact on households with children

**Fig. 5:**
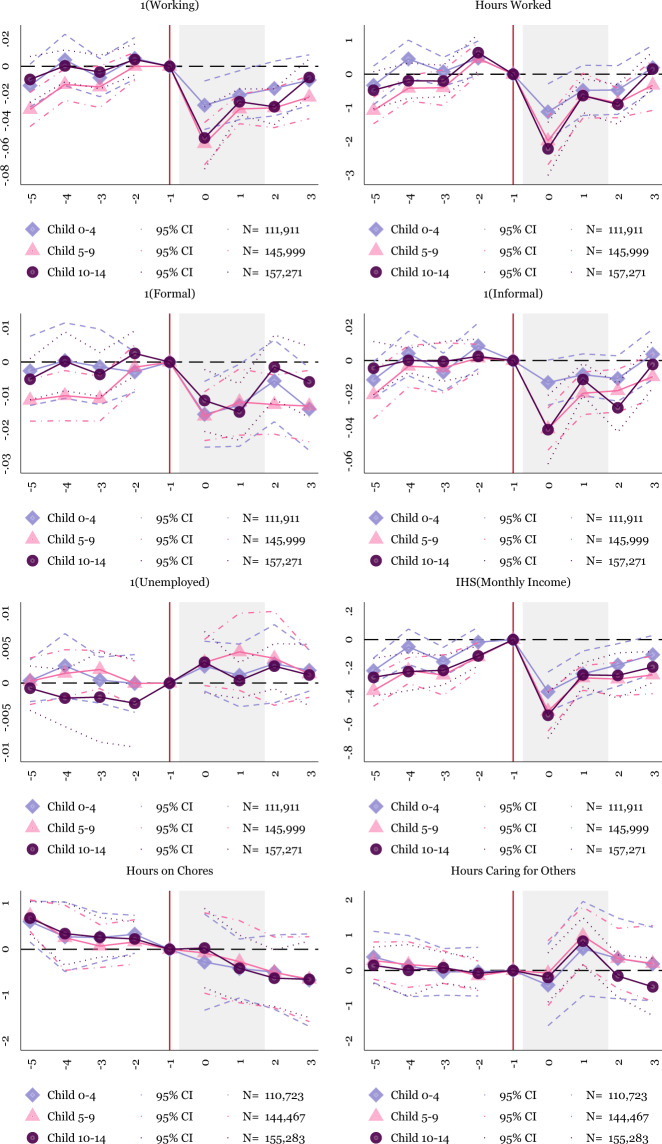
Event Study: Effect for Mothers (Household Heads or Spouses) by the Age of the Children Present in the Household. NOTES: OLS coefficients reported from Eq. (1). Baseline fixed effects include state-level fixed effects. Controls include indicators for the individual’s age and education. Sample weights are applied using the ENOE’s specified sample weights. The periods before −1 include 2019Q1–2019Q4. The omitted period represents 2020Q1, represented by the vertical line. There is no 2020Q2, so the post periods represent 2020Q3–2021Q2. The shaded period represents quarters in 2020. The main sample includes individuals who are 18–64. We also subset to household heads and their spouses with children present in the household. The above graph splits this sample into mothers with children under five, from five to nine, and ten to fourteen. Robust standard errors are clustered at the state level. Individual-level data from the traditional face-to-face National Occupation and Employment Survey (ENOE) 2019Q1–2020Q1 and 2020Q3–2021Q2

**Fig. 6:**
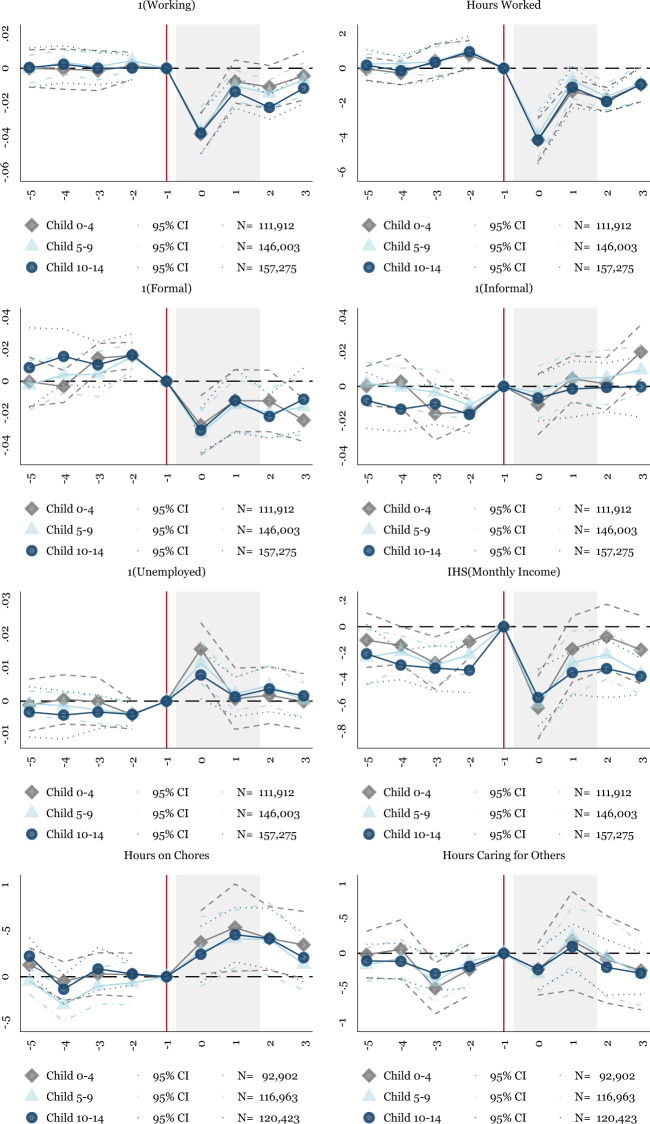
Event Study: Effect for Fathers (Household Heads or Spouses) by the Age of the Children Present in the Household. NOTES: OLS coefficients reported from Eq. (1). Baseline fixed effects include state-level fixed effects. Controls include indicators for the individual’s age and education. Sample weights are applied using the ENOE’s specified sample weights. The periods before –1 include 2019Q1–2019Q4. The omitted period represents 2020Q1, represented by the vertical line. There is no 2020Q2, so the post periods represent 2020Q3–2021Q2. The shaded period represents quarters in 2020. The main sample includes individuals who are 18–64. We also subset to household heads and their spouses with children present in the household. The above graph splits this sample into fathers with children under five, from five to nine, and ten to fourteen. Robust standard errors are clustered at the state level. Individual-level data from the traditional face-to-face National Occupation and Employment Survey (ENOE) 2019Q1–2020Q1 and 2020Q3–2021Q2

We next focus on households with children, especially those with school-aged children and younger children. Of particular interest is whether the primary sample of households fails to reallocate their time toward caring for others due to heterogeneous impacts by the presence of children. Households with children should be the most affected by school closures, and parents likely bear the main burden of this change. We split the sample into household heads and their spouses with children of different ages. We categorize children into under five, from five to nine, and ten to fourteen. Figure 5 shows results for mothers by the child age categories, and Fig. 6 presents the same for fathers.

In Fig. 5, mothers of school-aged children experience a one-quarter increase in their time caring for others. This increase in time spent caring occurs across mothers with children aged five to nine and ten to fourteen. The increase in time spent caring for others does not increase significantly for mothers of children under age five, even though the coefficient indicates a rise in time spent caring for others. For fathers in Fig. 6 hours on the house increase significantly and persistently, while time spent caring for others does not significantly increase.

While these results align more with our initial expectation than the baseline results, the failure of mothers to persistently increase time spent caring for others continues to be surprising. We attribute the relatively flat caring burden to women’s lower initial labor supply (OECD, 2020). In Mexico, women have lower pre-pandemic employment rates than high-income settings (Arceo-Gomez and Campos-Vazquez, 2010), indicating that prior to the pandemic, mothers already likely spent a substantial amount of their time on the household rather than in the labor market. There is, therefore, less ability for women’s time use to change during the pandemic relative to the United States and other high-income countries. Due to women’s lower labor supply generally, mothers in Mexico may be better positioned to absorb the school closures without an adjustment in time allocation. Extending from this primary explanation, past research has shown that women face barriers to childcare in Mexico (Ángeles et al., 2011, Calderon, 2014, Mateo Díaz and Rodriguez Chamussy, 2013), suggesting that the majority of households with young children may have already been caring for children at home. This theory is consistent with Fig. 5, where women with children under five experience less clear reductions in employment and hours worked as compared to the baseline findings.

To analyze other heterogeneous effects that may exist, we further explore households with children in Section C. First, we consider the impact on nuclear households where time spent caring for others is likely to be most representative of hours spent caring for children. In non-nuclear families, caring may be allocated toward elderly or ill family members, which is especially relevant during a pandemic. Because we cannot separate the time individuals spend caring for sick, elderly, or children, nuclear households with children will be our closest approximation to actual time spent caring for children. Restricting the sample to nuclear families also ensures that extended family members within the household are not sharing the caring responsibilities, which would change the interpretation of the main results. To consider nuclear families, we limit the sample of households to those composed of a head, the head’s spouse, and children of the household head, with mothers shown in Fig. C.1 and fathers presented in Fig. C.2.

The findings with nuclear families in Figs. C.1 and C.2 align with the results by child age, especially the findings for children 6–15. In households with school-aged children, women briefly increase their time caring for others, with the rise in time spent caring appearing in 2020Q4 and then dissipating. The effect only appears in households with school-aged children, and women do not significantly increase their time spent caring in households with very young children (under age five). Women with young children (under five) may be less likely to work before the pandemic began.

Second, we examine the impact on single-parent-headed households. We define single-parent headed households as households with only the household head and no spouse, where the household also has children under the age of 15. Figure C.3 shows the results for these households. In this sample of single parents, fathers fail to reallocate their time toward the household. Single mothers briefly increase their time spent caring for others, but the effect is only weakly significant.

Third, we show the impact on extended family members (non-household heads or spouses) who are present in households with children under the age of 15 in Fig. C.4. Here extended family members show no clear reallocation of time toward caring. Though, as in the baseline results, men increase their time on household chores. Fourth, we show the effects on households with children under 15 for all family members in Fig. C.5. Here the results are similar to the baseline, where men increase their time spent on the house, and there is little clear increase in time spent caring for others.

Overall, these results suggest that mothers with school-aged children increase their time caring for others for one period, 2020Q4, but the effect is temporary and does not persist. There is less evidence that mothers with young children reallocate their time toward caring, likely due to a high caring burden for this sample before the start of the pandemic. In almost all samples, aside from single-parent households, men increase their household chores but not their allocation of time toward caring for others.

### Heterogeneous effects

We also investigate heterogeneity in the treatment effects by marital status, urban status, states with a high HDI, age, and education in Section D. First, we consider whether married and unmarried adults respond differently to the onset of the pandemic in Fig. D.1. For men, the results mostly do not vary by marital status. The one exception to this finding is that married men experience a reduction in formal sector employment. Single men and women suffer comparable losses in formal employment, with married women experiencing the smallest loss.

In Fig. D.1, we see that differences between married and single women are larger relative to differences between married and single men. Stated differently, marital status is more pertinent to the labor market responses of women relative to men. For example, married women show smaller employment losses than single women. Married women also fail to reallocate their time to the household, while single women temporarily increase time on household chores. Married women also show a temporary spike in time caring for others. This last finding aligns with the results from households with children, suggesting that married women with children show a slight temporary increase in time spent caring for others. However, this effect is muted in the full sample of women.

Second, we examine differences by gender across urban and rural areas in Fig. D.2. For the urban/rural divide, the results are similar, except for two points. First, urban men experience larger employment losses on the extensive margin than rural men. Second, urban men and women are more likely to be unemployed than their rural counterparts.

Third, we divide Mexican states by their Human Development Index (HDI), where we separate states into above and below Mexico’s national HDI. We expect states with a higher HDI to be better equipped to respond to the pandemic and potentially have more opportunities for remote work. The results are presented in Fig. D.3. The results are similar across the sample, except that women in high HDI states increase their time spent caring for others in 2020Q4. These findings suggest that women in high-income states reallocate their time toward caring for others, potentially reflecting distinct labor supply norms.

Fourth, we show the impact by age in Fig. D.4 for women and Fig. D.5 for men. For women, the oldest group (55–70) shows the least recovery of employment and no increase in unemployment. For men, younger men (18–25) recover the fastest on both the extensive and intensive margins, mostly due to gains in the informal sector. Those who are 55–70 show the least gains in employment, similar to women.

Fifth, we present the results by education levels of women in Fig. D.6 and men in Fig. D.7. For men, the impact is similar across education levels. Women with higher education experience the least loss of employment overall and show almost no increase in reported unemployment.

### Does the ENOE capture the full impact of the pandemic? Alternative findings using the ETOE

#### ETOE event-study specification

Due to the fact that the ENOE fails to follow individuals through the entire course of the pandemic, we supplement our main findings from the ENOE using the ETOE. The ETOE has two main limitations. First, it was administered via a telephone survey and only represents households with access to a telephone. Second, the ETOE is a smaller sample than the traditional ENOE. While the documentation associated with the ETOE emphasizes that the ETOE is representative at the national level, the ETOE may exhibit bias for particular subsamples and outcomes. Still, despite these limitations, the ETOE also has two advantages. First, the ETOE provides the sole source of information on the impact of the pandemic lockdown on households. Second, the ETOE follows the same individuals over time, allowing us to consider the effect on individuals while controlling for time-invariant characteristics of individuals (individual fixed effects). We thus consider the ETOE to complement our main analysis using the traditional ENOE.

We present the ETOE findings using an event-study design similar to our baseline in Eq. (1). The primary adjustment is that the ETOE follows individuals over months rather than quarters in the post-pandemic period. Thus our focus is on 2019Q2–2020Q1 (four quarters before) and 2020M4–2020M11 (8 months after the pandemic). More formally, our ETOE event-study specification appears as:

2 $${Y}_{it}={\alpha }_{i}+\mathop{\sum }\limits_{Q=-4}^{7}{\beta }_{Q}\,{{{\mbox{COVID}}}}_{Q}+{{{\bf{X}}}}^{\prime} \gamma +{\epsilon }_{it}$$

where we follow individuals before and after the pandemic, with the event-study indicator variable, COVID_*Q*_. In this event study, *Q* represents the period relative to *Q* = 0, which captures the onset of the pandemic in 2020M4. *Q* ranges from four quarters before to 8 months after the start of the pandemic. We exclude the quarter before the beginning of the pandemic, *Q* = −1, which represents 2020Q1, as the baseline period. We also include individual fixed effects as *α*_*i*_, as the ETOE follows the same individuals over time. Due to the individual fixed effects and smaller sample size, we revise our controls to include age and age-squared. *ϵ*_*it*_ represents the standard error, which we cluster at the individual level. As with Eq. (1), we do not include time fixed effects as there is no variation in timing within each event-study indicator.

#### ETOE results

**Fig. 7:**
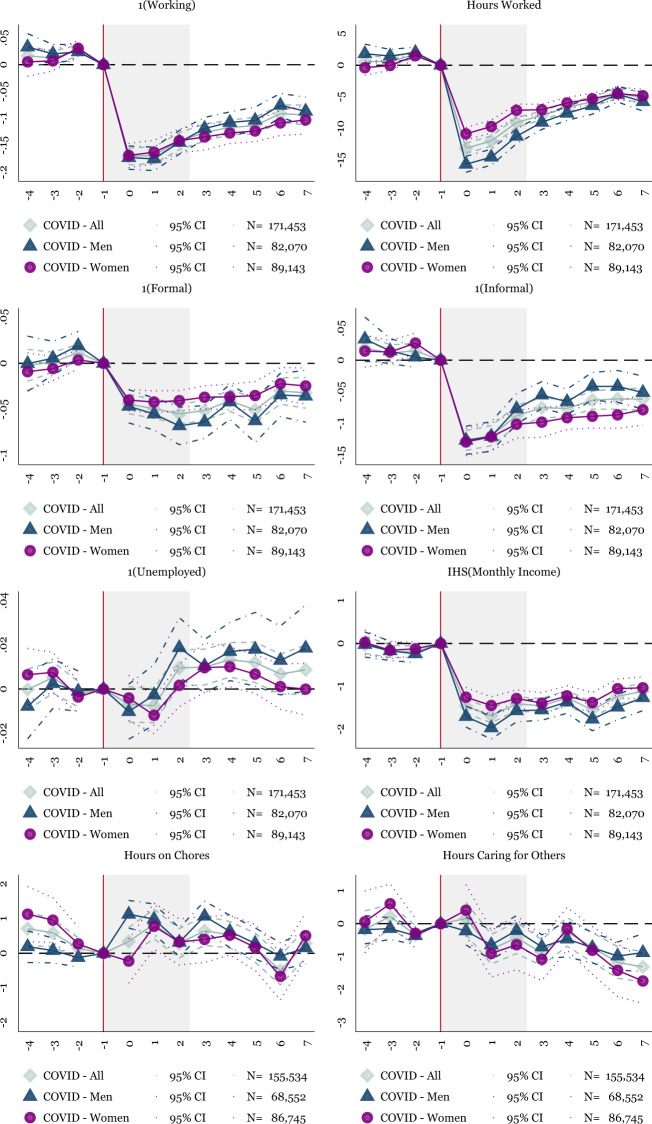
Event Study: Impact of the Initial Pandemic using the ETOE, Adults 18–64. NOTES: OLS coefficients reported from Eq. (2). Baseline fixed effects include individual fixed effects. Controls include the individual’s age and age-squared. Sample weights are applied using the ENOE’s specified sample weights. The plotted points show the quarters leading up to the COVID-19 recession. The post-periods represent months. The shaded areas show the lockdown duration of the sample (March 23rd–May 30th). The main sample includes individuals who are 18–64. Robust standard errors are clustered at the state level. Data includes National Occupation and Employment Survey (ENOE) 2019–2020Q1. For the post-period, April-November 2020, data is from the Encuesta Telefónica de Ocupación y Empleo (ETOE) 2020

Figure 7 shows the results for men (navy triangles), women (purple circles) as well as in aggregate (light blue diamonds). The vertical line represents the excluded period (2020Q1). The gray shaded area represents the three months in the *lockdown quarter* of the pandemic (2020Q2). The lockdown quarter is of particular interest, as these 3 months are not included in the traditional ENOE.

As anticipated (based on Fig. 2), the results using the ETOE suggest a more substantial impact on labor supply than the traditional ENOE. Beginning with extensive margin employment, the initial reduction in employment appears similar for men and women, a reduction in the probability of working by 17 percentage points. Both men’s and women’s employment starts to rebound by the 3 month of the pandemic, which continues to month seven, where the recovery stalls. Men’s employment recovers faster than women’s employment during months five through seven, similar to the results shown in the traditional ENOE.

Despite the similar changes on the extensive margin, the intensive margin hours worked declines by more for men than women. Men’s hours worked falls by 15 h, while women’s drops by 10 h. However, men’s hours worked quickly starts to rebound, and by the end of the ETOE series, men and women experience similar losses in intensive margin employment.

Turning to employment losses by sector, formal employment fails to recover, similar to the traditional ENOE. By contrast, informal employment declines substantially in the first quarter of the pandemic for both men and women, but men began to recover their informal employment faster than women. As seen in the main results, the informal sector in Mexico leads the recovery during the pandemic.

In the bottom two panels of Fig. 7, men compensate for their employment losses by spending more time on household chores at the start of the pandemic. This spike in household chores is apparent during the lockdown phase of the pandemic but then starts to decline by month three as men return to work. Women also briefly increase their time on household chores in the second month of the pandemic. There is no clear reallocation toward time spent caring for others, for men or women.

Put together, the dynamic effects displayed in the ETOE results show a similar pattern to the traditional ENOE. Two exceptions exist. First, the ETOE results suggest a much larger magnitude of employment loss for households, likely because the ETOE sample includes the lockdown phase of the pandemic. Second, in the ETOE, both men and women briefly increase their time spent on household chores. In the main findings, men’s reallocation of time toward household chores continues for the entire sample, while women show no apparent response. The differences for men’s time use may reflect sample differences or methodological differences due to the use of individual fixed effects in the ETOE analysis.

## Robustness

### Empirical specification

In addition to the event study, we also show the effect of the pandemic, on average, over the post-pandemic period using a grouped post-period specification. Here we compare the effect of the pandemic over 2020Q3–2021Q2 to pre-pandemic periods, 2019Q1–2020Q1.

More specifically, we estimate the labor supply and time use of individual *i* during time *t* as:

3 $${Y}_{ist}={\alpha }_{s}+\beta \,{{{\mbox{COVID-19 Pandemic}}}}_{t}+{{{\bf{X}}}}^{\prime} \gamma +{\tau }_{T}+{\epsilon }_{ist}$$

where *Y*_*ist*_ is the outcome of interest and includes labor supply, income, and time use. COVID-19 Pandemic_*t*_ is an indicator that takes the value of one for the post-pandemic periods, 2020Q3, 2020Q4, 2021Q1, and 2021Q2. COVID-19 Pandemic_*t*_ is equal to zero for quarters 2019Q1–2020Q1. The remainder of Eq. (3) is similar to Eq. (1), except we include *τ*_*T*_, which captures quarterly fixed effects.

### Results

We show the severity of the COVID-19 recession for all adults in Table 2, with women in Panel A and men in Panel B. The results reflect the findings from Eq. (3) and give the average impact over the post period (2020Q3–2020Q2).

**Table 2:** Grouped Post-period: Adult employment, income, and time use

	1(Working)	Hours worked	1(Formal)	Formal hours	1(Informal)	Informal hours	Hours chores	Hours caring	IHS of Income
	(1)	(2)	(3)	(4)	(5)	(6)	(7)	(8)	(9)
*Panel A: Women*									
1(COVID-19 Pandemic)	−0.034^***^	−1.557^***^	−0.012^***^	−1.221^***^	−0.022^***^	−0.128	−0.439^**^	−0.063	−0.152^***^
	(0.003)	(0.176)	(0.002)	(0.215)	(0.002)	(0.287)	(0.201)	(0.181)	(0.040)
Observations	1,045,424	1,045,424	1,045,424	268,245	1,045,424	271,756	1,016,126	1,016,126	1,045,424
Adjusted R-squared	0.071	0.050	0.158	0.082	0.037	0.020	0.157	0.096	0.046
Pre-Mean Dependent	0.500	18.526	0.223	41.671	0.277	33.283	20.933	7.416	3.076
Post-Mean Dependent	0.469	17.066	0.215	40.331	0.254	33.066	20.507	7.315	2.943
*Panel B: Men*									
1(COVID-19 Pandemic)	−0.030^***^	−2.309^***^	−0.027^***^	−1.062^***^	−0.003	−1.132^***^	0.372^***^	0.014	−0.166^**^
	(0.004)	(0.343)	(0.003)	(0.295)	(0.003)	(0.320)	(0.083)	(0.076)	(0.076)
Observations	943,685	943,685	943,685	404,364	943,685	367,956	741,891	741,891	943,685
Adjusted R-squared	0.126	0.102	0.170	0.047	0.155	0.036	0.031	0.041	0.109
Pre-Mean Dependent	0.835	37.645	0.391	47.058	0.443	43.374	4.928	2.081	5.331
Post-Mean Dependent	0.807	35.356	0.371	45.912	0.436	42.043	5.278	2.142	5.184
Quarter FE	X	X	X	X	X	X	X	X	X
State FE	X	X	X	X	X	X	X	X	X
Controls	X	X	X	X	X	X	X	X	X

OLS coefficients reported from Eq. (3). The coefficient indicates the COVID-19 pandemic recession, which equals one for periods 2020Q3 and after. Baseline fixed effects include quarter fixed effects and state-level fixed effects. Controls include indicators for the individual’s education and the individual’s age. Sample weights are applied using the ENOE’s specified sample weights. The main sample includes individuals who are 18–64. Robust standard errors are clustered at the state level and are reported in parentheses. ***, ** represent statistical significance at 1, 5, and 10% levels. Individual-level data from the traditional face-to-face National Occupation and Employment Survey (ENOE) 2019Q1–2020Q1 and 2020Q3–2021Q2

We find that at both the intensive and extensive margins, employment declines during the COVID-19 pandemic. Men’s employment declines by 3% and women’s employment falls by 3.5%. For formal employment, the declines in employment are slightly different across men and women-formal employment declines by 2.7% for men, which is larger than the 1.2% decline that women experience. Men also appear to shift into informal work to compensate for formal employment losses. Men’s informal employment shows no difference post-pandemic. For women, informal employment is 2.2% lower in the post-pandemic period.^12^

Considering time use of men and women, the results are similar to the baseline event-study results. Men increase their time in household chores, but women do not. Neither men nor women increase their time caring for others (including children). These findings suggest that the burden of household work appears to be absorbed mostly by men. There is no similar shift toward time spent with children (or caring in general) for men or women.

For the specification with the grouped post-period, we also show the findings subsetting to parents with at least one school-aged child (5–14) in Table B.6 and at least one child under five in Table B.7. In this subsample of parents, mothers fail to increase their time spent caring for others. Similarly, their household chores decline (on average) over the post-pandemic period. Fathers instead make up for the decline in household chores and increase their time spent on chores by 0.4 h.

From the results presented in Table B.6 and Table B.7, the pre-pandemic means also reveal the substantial amount of time that mothers already spent on the household. Mothers with young children (under five) spent 45 h per week pre-pandemic on caring time and household chores. Women with school-aged children spent slightly less but still spent 38 h in total on household chores plus caring for others. This substantial amount of time devoted to the household and children helps explain why mothers do not noticeably reallocate their time during the pandemic. Mothers in this sample are already dedicating their primary time to the household.

Further, we show the measures of time use for mothers and fathers broken into all other categories of time use in Tables B.8 and B.9. Fathers mainly increase their time on chores (from the main results), household maintenance, and time spent building. Mothers decrease their time on total household production (chores, maintenance, building, purchasing, and transporting) by more than 2 h. The largest reduction for mothers comes from hours spent transporting other family members.

Put together, we find that women do not shift their time toward caring for others or the household (on average) during the pandemic and only briefly reallocate their time in the event-study specification (for a single quarter). This reallocation of time toward children only occurs for mothers in households with school-aged children (as opposed to young children). However, mothers were already spending a significant portion of their time caring for children, especially young children, which helps explain why mothers do not show marked changes in time allocation during the pandemic. Mothers also substantially reduce their time spent on household production, while fathers increase their time spent on household production.

### Alternative results

To test whether the results are robust over different specifications, we show alternative forms of Eq. (3) in Fig. A.2. First, we plot the results adding individual fixed effects. Then, we show the results altering the baseline specification with state fixed effects by removing quarter fixed effects, removing controls, and adding state-level trends. In the bottom three specifications, we also show the baseline specification for mothers and fathers with children under age five, 5–9, and 10–14. Here the takeaways generally reflect the baseline results, except the magnitude of the effect does adjust depending on the chosen specification.

## Additional findings

### Intra-household changes in time use and income

Labor supply decisions are not made independently of one’s partner. Therefore, we examine relative changes in labor supply and time use across couples in response to the pandemic. We select a subsample of heterosexual couples consisting of one man and one woman as well as at least one child under age 15. We calculate the woman’s relative contribution to household production, income, and labor supply among these couples. All measures are bounded between zero and one by construction, with higher values indicating the woman contributing relatively more. For households with no income, we assign a share of one-half to the woman. We do the same when both members of the couple have zero values for any of our other outcomes of interest.

We present the results in Table 3. For each outcome variable, we first report the results where we drop any household where both members have zero values for the outcome of interest. These results are presented in odd-numbered columns. We then replace zeros with contributions of one-half in even-numbered columns, which are our preferred estimates. Looking first at employment and income measures, each suggests little change in the wife’s contribution to employment or income.

**Table 3:** Intra-household inequality: Share contributed by the wife in households with a husband, wife, and children

	Share hours worked		Share income		Share hours chores		Share hours caring	
	(1)	(2)	(3)	(4)	(5)	(6)	(7)	(8)
1(COVID-19 Pandemic)	−0.001	0.004	−0.002	0.003	−0.016^***^	−0.018^***^	−0.002	−0.009^**^
	(0.003)	(0.003)	(0.002)	(0.003)	(0.003)	(0.003)	(0.003)	(0.003)
Observations	255,082	270,531	212,293	270,531	265,913	270,531	174,182	270,531
Adjusted R-squared	0.057	0.051	0.071	0.083	0.049	0.049	0.051	0.052
Pre-Mean Dependent	0.198	0.213	0.209	0.278	0.882	0.877	0.828	0.710
Post-Mean Dependent	0.199	0.219	0.208	0.280	0.867	0.860	0.824	0.701
Quarter FE	X	X	X	X	X	X	X	X
State FE	X	X	X	X	X	X	X	X
Controls	X	X	X	X	X	X	X	X
Including Zeros		X		X		X		X

OLS coefficients reported from Eq. (3). The coefficient indicates the COVID-19 pandemic recession, which equals one for periods 2020Q3 and after. Baseline fixed effects include quarter fixed effects and state-level fixed effects. Controls include indicators for the individual’s education and the individual’s age. Sample weights are applied using the ENOE’s specified sample weights. For this specification, the sample only includes households with one husband, one wife and at least one child. Women must be 18–64 in order to be included in the sample. Shares excluding zeros are based on reported information in the ENOE. Including zeros means that zeros are replaced with 50-50 shares. Robust standard errors are clustered at the state level and are reported in parentheses. ***, ** represent statistical significance at 1, 5, and 10% levels. Individual-level data from the traditional face-to-face National Occupation and Employment Survey (ENOE) 2019Q1–2020Q1 and 2020Q3–2021Q2

We next examine time use measures in Columns (5)–(8) of Table 3. These results show that women spend less relative time on the household, both including and excluding zeros. For hours spent caring for others, the findings suggest a reduction in time spent caring when zeros are included. These results indicate that Mexican households did not shift the general caring burden to women during the pandemic. Instead, men appear to increase their time spent on household production. However, a limitation of this approach is that we cannot separate caring for children from general caring for other individuals (sick and elderly).

### Effects on children

We conclude our study by considering the effects of the pandemic recession on children. We are particularly interested in testing whether children reduced their time spent on schoolwork as well as their school enrollment. In our analysis, we include all children who are age 6–16. We select children 6–16 as upper secondary school (targeting 15 years of school) is mandatory in Mexico (UnoiNews, 2012).

In Panel A of Table 4, our results demonstrate that children slightly reduced both their enrollment and their time on schoolwork. In Column (1), the probability of being enrolled in school falls by one percentage point in the post-pandemic periods. In Column (2), children reduced their school activities by 8 h per week in response to the pandemic. The reduction is more than 25% of the pre-pandemic mean. When we look at children separately by gender in Panels B and C, we see slightly larger reductions for boys relative to girls. Overall, the results are consistent with our findings for parental time use and suggest that the parents did not fully compensate for the school closures with homeschooling activities.

**Table 4:** Grouped post-period: Effects on children 6–16

	1(In school)	Hours school	1(Working)	Hours worked	Hours chores	Hours caring
	(1)	(2)	(3)	(4)	(5)	(6)
*Panel A: All*						
1(COVID-19 Pandemic)	−0.010^***^	−8.780^***^	0.004^**^	0.308^***^	0.501^***^	0.144^***^
	(0.002)	(0.457)	(0.002)	(0.062)	(0.092)	(0.039)
Observations	608,035	225,117	608,035	608,035	271,139	271,139
Adjusted R-squared	0.236	0.216	0.122	0.111	0.093	0.014
Pre-Mean Dependent	0.942	32.296	0.052	1.450	5.988	0.784
Post-Mean Dependent	0.937	23.864	0.056	1.728	6.534	0.929
*Panel B: Girls*						
1(COVID-19 Pandemic)	−0.010^***^	-8.508^***^	0.001	0.134^**^	0.677^***^	0.245^***^
	(0.002)	(0.465)	(0.002)	(0.057)	(0.131)	(0.056)
Observations	296,979	111,804	296,979	296,979	135,511	135,511
Adjusted R-squared	0.222	0.220	0.066	0.055	0.154	0.024
Pre-Mean Dependent	0.947	32.341	0.030	0.747	7.566	1.105
Post-Mean Dependent	0.942	24.258	0.032	0.858	8.299	1.336
*Panel C: Boys*						
1(COVID-19 Pandemic)	−0.010^***^	−9.059^***^	0.006^***^	0.428^***^	0.400^***^	0.055^*^
	(0.002)	(0.460)	(0.002)	(0.078)	(0.078)	(0.032)
Observations	311,056	113,313	311,056	311,056	135,628	135,628
Adjusted R-squared	0.249	0.214	0.180	0.165	0.070	0.007
Pre-Mean Dependent	0.938	32.250	0.072	2.127	4.381	0.458
Post-Mean Dependent	0.932	23.477	0.080	2.552	4.774	0.524
Quarter FE	X	X	X	X	X	X
State FE	X	X	X	X	X	X
Controls	X	X	X	X	X	X

OLS coefficients reported from Eq. (3). The coefficient indicates the COVID-19 pandemic recession, which equals one for periods 2020Q3 and after. Baseline fixed effects include quarter fixed effects and state-level fixed effects. Controls include indicators for the individual’s education and the individual’s age. Sample weights are applied using the ENOE’s specified sample weights. Sample includes children who are 6–16. Robust standard errors are clustered at the state level and are reported in parentheses. ***, **, * represent statistical significance at 1, 5, and 10% levels. Individual-level data from the traditional face-to-face National Occupation and Employment Survey (ENOE) 2019Q1–2020Q1 and 2020Q3–2021Q2

Two crucial features of Mexico’s public education policy are essential to contextualize the results. First, Mexico had one of the most prolonged school closures globally in terms of the number of days, with over 217 days of school closures. Second, rather than only having remote online schooling, Mexico also televised learning activities, even though most students used smartphones for their schooling activities. However, our data do not allow us to discern among the different distance learning techniques: online versus televised.

Offsetting the reduction in school enrollment and time on school, children also increase their employment and hours worked. The effect is more noticeable for boys than girls. Children also increase their time spent on the household and time caring for others. The increase in household production for children is roughly equivalent to men’s increase in household production, one-half of an hour. Children are the only group that consistently increases their time spent caring for others, suggesting that children may have absorbed some of the caring burden associated with the pandemic.

## Conclusion

Our results suggest that the COVID-19 pandemic severely impacted Mexican households. During the initial onset of the pandemic, employment fell by 17 percentage points for both men and women. After the initial downturn, men’s employment recovered faster than women’s. By the end of 2021Q2, men had almost completely recovered their initial employment levels on both the extensive and intensive margins. The majority of employment gains occurred in the informal sector, suggesting potential growth in precarious labor conditions.

Our findings also indicate that men contributed more to household chores during the pandemic. For women, the only noticeable change in time use is a temporary increase in time spent caring for others by mothers with school-aged children (during 2020Q4). However, women do not generally shift their time use, and the increase for mothers only occurs temporarily and does not hold on average over the duration of the pandemic period. We attribute the small change in time use to the fact that mothers in Mexico were already spending a significant amount of time caring for others and on household chores pre-pandemic. These findings are confirmed when we consider intra-household dynamics for couples. Within-household, women appear to decrease their time spent on household chores, but time spent caring for others is constant or declines. Though, we caution that mothers’ time spent on household chores was falling before the pandemic, making the actual effect of the pandemic difficult to disentangle.

To better contextualize the household response to the pandemic, we also measure changes in children’s time use during the pandemic. While children did not change their school enrollment, they did reduce their time spent on schoolwork by 25% from the pre-pandemic mean. These findings corroborate our parental time use results and suggest that parents fail to (observably) compensate for the lost instructional time. Together, our results indicate that the pandemic’s true burden may then be on children, and future research should consider the detrimental effects of lost school time. Ideally, public policies would direct support toward remediating the adverse effects on children. These child-focused challenges are unique to the pandemic recession relative to previous economic downturns.

## Supplementary information

Online Appendices

## Footnotes

1 A firm is considered formal based on accounting practices in Mexico, including filing incorporation to Mexico’s public registration authority and paying taxes to the federal and local governments. On the labor side, formal employment is defined as contributing to Mexico’s social security system, including having access to health insurance and participating in the pension system (Alvarez and Ruane, 2019).

2 Other related work in high-income countries includes (Mark, Hurst and Karabarbounis, 2013, Alon et al., 2020b, Bartik et al., 2020, Boca et al., 2020, Cajner et al., 2020, Czymara et al., 2020, Farré et al., 2020, Heggeness, 2020, Prados and Zamarro, 2020, Yamamura and Tsustsui, 2021, Zamarro and Prados, 2021).

3 At the same time, other portions of the literature highlight the impact of the pandemic on fathers. Leukhina and Yu (2020) finds that household production increased for both married men and women with children during the COVID-19 pandemic. Further, Lucia, Piccoli and Pieroni (2021) finds that fathers’ involvement in homeschooling and childcare was crucial in Italy.

4 While schools are generally open as of December 2021, the degree of re-opening still varies across Mexican states.

5 The aggregate figures mask considerable heterogeneity within a given country. For example, certain regions in the United States remained in-person during the 2020–2021 academic year, while others were entirely online.

6 During the pandemic, 28.6% of all households with children or young adults (3–29 years old) bought smartphones, 26.4% hired wireless home internet service, 14.3% purchased a computer or laptop, 6.2% acquired mobile internet coins, 5.2% bought a digital TV, and 5.1%, a tablet. Further, students in primary school (1st–6th grade) used smartphones the most (70.2%), followed by laptops (9.8%), TVs (6.7%), tablets (5.6%), and computers (3.9%). Similarly, students in middle school (7th–9th grade) utilized smartphones the most (68.5%), followed by laptops (17.4%), computers (8.1%), tablets (3.0%), and TVs (1.8%). Also, students in high school (10th–12th grade) employed smartphones the most (53.3%), followed by laptops (30.9%), PCs (13.1%), tablets (2.2%), and TVs (0.2%).

7 Note that the ECOVID-ED is a special cross-sectional survey and does not allow us to infer anything about the *changes* in parental involvement after the start of the pandemic.

8 We exclude December 2020 through March 2021 as the sample size shrinks.

9 If an individual reports any time-use measure, they are included in the time-use sample. If individuals report no measure of time use, we replace all their time use values with missing rather than zeros. If an individual reports one measure of time use but not another, we assume the categories that are left blank indicate zero hours on that activity.

10 We also show time on total household tasks, hours house, which includes time on household maintenance, time on household chores, time building and maintaining the house, and time purchasing.

11 Throughout the analysis, we use ENOE’s definition of education. The ENOE defines education levels as “ninguno” or none, “preescolar” or less than primary, “primaria” or primary, “secundaria” or middle, “preparatoria o bachillerato” or high school, “carrera técnica” or technical education, “profesional” or professional, “maestría” or masters, and “doctorado” or doctorate. Adults with education levels of “no sabe” or unknown/missing are omitted.

12 For hours worked, we use the baseline value of hours worked specified by the ENOE, which includes zeros. For formal employed hours and informal employed hours, we take the hours worked for individuals who report either formal or informal employment. Thus, we effectively condition hours worked in columns (4) and (6) on individuals reporting employment in the formal or informal sector.

## References

[CR1] Adan, S. M., Hoehn-Velasco, L., & Balmori de la Miyar, J. R. (2020). Great Lockdown vs. Great Recession: Is This Time Different for the Labor Market?. *Journal of Economics and Business*.

[CR2] Agency, CIA Central Intelligence, (2017). *The World Factbook 2017*, in “in” CIA Washington, DC.

[CR3] Aguiar M, Hurst E, Karabarbounis L (2013). Time use during the great recession. American Economic Review.

[CR4] Albanesi S, Kim J (2021). Effects of the COVID-19 Recession on the US Labor Market: Occupation, Family, and Gender. Journal of Economic Perspectives.

[CR5] Arceo-Gomez, E. & Campos-Vazquez, R. (2010). *Labor Supply of Married Women in Mexico: 1990–2000*, Technical Report, El Colegio de México, Centro de Estudios Económicos.

[CR6] Azevedo JP, Hasan A, Goldemberg D, Geven K, Iqbal SyedahAroob (2021). Simulating the potential impacts of COVID-19 school closures on schooling and learning outcomes: A set of global estimates. The World Bank Research Observer.

[CR7] Bansak C, Starr M (2021). COVID-19 shocks to education supply: How 200,000 US households dealt with the sudden shift to distance learning. Review of Economics of the Household.

[CR8] Bartik, A. W., Bertrand, M., Lin, F., Rothstein, J., & Unrath, M. (2020). *Measuring the labor market at the onset of the COVID-19 crisis*, Technical Report, National Bureau of Economic Research.

[CR9] Bellemare MF, Wichman CJ (2020). Elasticities and the inverse hyperbolic sine transformation. Oxford Bulletin of Economics and Statistics.

[CR10] Bustelo, M., Flabbi, L., Piras, C., & Tejada, M. (2019). *Female labor force participation, labor market dynamic, and growth*, Technical Report, IDB Working Paper Series.

[CR11] Cajner, T., Crane, L. D., Decker, R. A., Grigsby, J., Hamins-Puertolas, A., Hurst, E., Kurz, C., & Yildirmaz, A. (2020). *The U.S. Labor Market during the Beginning of the Pandemic Recession*, Working Paper 27159, National Bureau of Economic Research May.

[CR12] Claudia, G. (1994). *The U-shaped Female Labor Force Function in Economic Development and Economic History*. Technical Report, National Bureau of Economic Research.

[CR14] Croda E, Grossbard S (2021). Women pay the price of COVID-19 more than men. Review of Economics of the Household.

[CR15] Czymara, C. S., Langenkamp, A., & Cano, T. (2020). Cause for concerns: gender inequality in experiencing the COVID-19 lockdown in Germany*. European Societies*, 1–14.

[CR16] Daniela Del, B., Oggero, N., Profeta, P., & Rossi, M. (2020). *Women’s work, housework and childcare, before and during COVID-19*. 10.1007/s11150-020-09502-1 PMC7474798 32922242

[CR13] de Córdoba, J. & Montes, J. (2020). School in Mexico During Covid-19 Means Turning on the TV.

[CR32] de Hoyos, R. (2021). Por qué no abrimos las escuelas?. *Nexos*.

[CR17] Dingel, J. I., & Neiman, B. (2020). How many jobs can be done at home?, Technical Report, National Bureau of Economic Research.

[CR18] Duman, A. (2020). Wage Losses and Inequality in Developing Countries: labor market and distributional consequences of Covid-19 lockdowns in Turkey*. Available at SSRN 3645468*.

[CR19] Evalúa, Cómo Vamos? (2020). México, Programas de Apoyo Económico Frente al COVID-19 en el Mundo. *México Evalúa*, June.

[CR20] Farzana, A., Dhillon, A., Roy, S., et al. (2021). The gendered crisis: livelihoods and mental well-being in India during COVID-19, Technical Report, World Institute for Development Economic Research (UNU-WIDER).

[CR21] Gabriela, C. (2014). *The effects of child care provision in Mexico*, Technical Report, Working Papers.

[CR22] Garrote Sanchez D, Parra NicolasGomez, Ozden C, Rijkers B, Viollaz M, Winkler H (2021). Who on Earth Can Work from Home?. The World Bank Research Observer.

[CR24] Gottlieb C, Grobovšek J, Poschke M, Saltiel F (2021). Working from home in developing countries. European Economic Review.

[CR25] Grewenig, E., Lergetporer, P., Werner, K., Woessmann, L., & Zierow, L. (2020). *COVID-19 and educational inequality: how school closures affect low-and high-achieving students*. Technical Report. 10.1016/j.euroecorev.2021.103920 PMC8474988 34602646

[CR26] Gustavo, A. Gadsden, P., Galiani, S., Gertler, P., Herrera, A., Kariger, P., & Seira, E. (2011). Evaluación de impacto del programa estancias infantiles para apoyar a madres trabajadoras, *Informe Final de la Evaluación* de Impacto. Instituto Nacional de Salud Pública, México.

[CR27] Hale, T., Webster, S., Petherick, A., Phillips, T., & Kira B. (2020). *Oxford COVID-19 Government Response Tracker [Internet]. Coronavirus Government Response Tracker. 2020*. 10.1038/s41562-021-01079-8 33686204

[CR28] Hans, D., Patzina, A., & Lerche, A. (2021). Social inequality in the homeschooling efforts of German high school students during a school closing period. *European Societies*, 23(sup1), S348–S369.

[CR29] Hasan SM, Rehman A, Zhang W (2021). Who can work and study from home in Pakistan: evidence from a 2018–19 nationwide household survey. World Development.

[CR30] Heggeness, M. L. (2020). Estimating the immediate impact of the COVID-19 shock on parental attachment to the labor market and the double bind of mothers. *Review of Economics of the Household*, 1–26. 10.1007/s11150-020-09514-x PMC7584481 33132792

[CR31] Hoehn-Velasco L, Penglase J (2021). Does unilateral divorce impact women’s labor supply? Evidence from Mexico. Journal of Economic Behavior & Organization.

[CR33] Huebener M, Sevrin W, Spiess CK, Nico A S, Gert G W (2021). Parental well-being in times of Covid-19 in Germany. Review of Economics of the Household.

[CR34] IMSS, (2020). Puestos de Trabajo Afiliados al Instituto Mexicano sel Seguro Social. *IMSS*, August.

[CR35] Jorge, J. & Ruane, C. (2019). *Informality and Aggregate Productivity: The Case of Mexico*.

[CR36] Kohara M (2010). The response of Japanese wives’ labor supply to husbands’ job loss. Journal of Population Economics.

[CR37] Kugler, David, M., Viollaz, M., Duque, D.V.A., Gaddis, I., Newhouse, D. L., Palacios-Lopez, A., Weber, M., et al. (2021). *How Did the COVID-19 Crisis Affect Different Types of Workers in the Developing World?*. Technical Report, The World Bank. 10.1016/j.worlddev.2023.106331 PMC10284455 37362609

[CR38] Leukhina, O. & Yu, Z. (2020). Home Production and Leisure During the COVID-19. *FRB St. Louis Working Paper*, (2020–025).

[CR39] Levy, S. (2010). *Good intentions, bad outcomes: Social policy, informality, and economic growth in Mexico*. Brookings Institution Press.

[CR40] Linda, F., Fawaz, Y., González, L., & Graves, J. (2020). *How the COVID-19 Lockdown Affected Gender Inequality in Paid and Unpaid Work in Spain*, Working Paper 13434, IZA July.

[CR41] Mangiavacchi L, Piccoli L, Pieroni L (2021). Fathers matter: Intrahousehold responsibilities and children’s wellbeing during the COVID-19 lockdown in Italy. Economics & Human Biology.

[CR42] Matías, B., Fazio, M., & Algazi, S. (2012). (In) formal and (un) productive: the productivity costs of excessive informality in Mexico.

[CR43] Matt, R. & Gallón, N. (2020). Mexico’s solution to the Covid-19 educational crisis: Put school on TV. Aug.

[CR44] Melanie, K., Prinz, D., Newhouse, D., Palacios-Lopez, A., Pape, U., & Weber, M. (2021). The Early Labor Market Impacts of COVID-19 in Developing Countries.

[CR45] Melinda, P. M. (2021). Where Are They Now? Workers with Young Children during COVID-19. *Federal Reserve Bank of Atlanta Policy Hub Working Paper*, 10.

[CR46] Mercedes, M. D., & Chamussy, L. R. (2013). Childcare and women’s labor participation: evidence for Latin America and the Caribbean.

[CR47] Mongey, S. & Weinberg, A. (2020). Characteristics of workers in low work-from-home and high personal-proximity occupations. *Becker Friedman Institute for Economic White Paper*.

[CR48] Nathan, B., Davis, C. A., López-Peña, P., Mitchell, H., Mushfiq Mobarak, A., Naguib, K., Emy Reimão, M., Shenoy, A., & Vernot, C. (2020). *Migration and the labour market impacts of covid-19*, Technical Report, WIDER Working Paper.

[CR49] Nora, L., Martinez-Pabon, V., Sanz, F., & Youngerue, S. (2020). *The Impact of COVID-19 lockdowns and expanded social assistance on inequality, poverty and mobility in Argentina, Brazil, Colombia and Mexico*, Technical Report 46, CEPR September.

[CR50] Novta, N. & Wong, J. (2017). *Women at work in Latin America and the Caribbean*. International Monetary Fund.

[CR51] Ortega-Díaz, A. (2020). Marital Status and Poverty with Gender Bias, in Advances in Women’s Empowerment: Critical Insight from Asia, Africa and Latin America. Emerald Publishing Limited.

[CR52] Peluffo C, Viollaz M (2021). Intra-household exposure to labor market risk in the time of Covid-19: lessons from Mexico. Review of Economics of the Household.

[CR53] Prados, M. & Zamarro, G. (2020). Gender Differences in Couples’ Division of Childcare, Work and Mental Health During COVID-19. *Review of Economics of the Household*, (003). 10.1007/s11150-020-09534-7 PMC7811157 33488316

[CR54] Psacharopoulos G, Tzannatos Z (1993). Economic and demographic effects on working women in Latin America. Journal of Population Economics.

[CR55] Raymundo, C. V., Esquivel, G., & Badillo, R. (2020). *How has labor demand been affected by the COVID-19 pandemic?* Evidence from job ads in Mexico, Technical Report 46, CEPR September.

[CR56] Saltiel F (2020). Who can work from home in developing countries. Covid Economics.

[CR57] Sandeep, M. (2020). Gender differentiated economic responses to crises in developing countries: insights for COVID-19 recovery policies. *Review of Economics of the Household*, 1–16. 10.1007/s11150-020-09512-z PMC7557150 33078059

[CR58] Sarah, B., Benzell, S., & Solares, R. (2020). *Ranking how national economies adapt to remote work*. MIT Sloan Management Review.

[CR59] Simone, S., Danquah, M., Osei, R. D., & Sen, K. (2021). The labour market impact of COVID-19 lockdowns: evidence from Ghana.

[CR60] Skoufias E, Parker SW (2006). Job loss and family adjustments in work and schooling during the Mexican peso crisis. Journal of Population Economics.

[CR61] Sophie, L., & Filho, N. M. (2021). Evaluating the Impact of the Covid Emergency Aid Transfers on Female Labor Supply in Brazil. *CENTRO*.

[CR62] Titan A., Doepke, M., Olmstead-Rumsey, J., & Tertilt, M. (2020). *The impact of COVID-19 on gender equality*, Technical Report, National Bureau of economic research.

[CR63] Titan, A., Doepke, M., Olmstead-Rumsey, J., & Tertilt, M. (2020). *This Time It’s Different: The Role of Women’s Employment in a Pandemic Recession*, Technical Report, National Bureau of Economic Research.

[CR64] UNICEF, et al. (2021). COVID 19 and school closures. *One year of education disruption. UNICEF*.

[CR65] UnoiNews, (2012). Obligatoria en México la educación media superior. Aug.

[CR66] USDE, (2021). Education in a Pandemic:The Disparate Impacts of COVID-19 on America’s Students. June.

[CR23] von Gaudecker, Hans-Martin, Holler, R., Janys, L., Siflinger, B., & Zimpelmann, C. (2020). Labour Supply During Lockdown and a. 10.1016/j.labeco.2021.102055 PMC8444357 34545270

[CR67] Vasavi, B., Grover, S., Sharma, A., et al. (2020). COVID-19 pandemic, lockdown and the Indian labour market: evidence from PLFS 2017-18, *Indira Gandhi Institute of Development Research*.

[CR68] Xavier, E., Sharma, M., Gupta, S., Birla, B., et al. (2020). *Impact of COVID-19 pandemic on labor Supply and gross value added in India*. *Available at SSRN*.

[CR69] Yamamura E, Tsustsui Y (2021). The impact of closing schools on working from home during the COVID-19 pandemic: evidence using panel data from Japan. Review of Economics of the Household.

[CR70] Zamarro G, Prados MJ (2021). Gender differences in couples’ division of childcare, work and mental health during COVID-19. Review of Economics of the Household.

[CR71] Zenyazen, F. (2020). México pierde 1.1 millones de empleos formales en lo que va de la pandemia*. El Financiero*, https://www.elfinanciero.com.mx/economia/mexico-pierde-1-1-millones-de-empleos-formales-en-lo-que-va-de-la-pandemia-segun-datos-del-imss.

