# Supplementary material for: The impact of the COVID-19 recession on Mexican households: evidence from employment and time use for men, women, and children
Source: Rev Econ Househ. 2022 Jan 29;20(3):763–97. doi: 10.1007/s11150-022-09600-2 (PMC8799985; doi:10.1007/s11150-022-09600-2)
Supplement: Supplementary file 1 — Online Appendices [file 11150_2022_9600_MOESM1_ESM.pdf]

Online Appendices

Contents

Contents 1

List of Figures 1

List of Tables 1

Appendix A Additional Figures 3

Appendix B Additional Tables 5

Appendix C Additional Effects by Households with Children 13

Appendix D Additional Heterogeneous Effects 18

List of Figures

A.1 Questionnaires for Time Use . . . 3
A.2 Alternative Specifications for the Grouped Post-period Results . . . 4
C.1 Effect for Mothers in Nuclear Families by the Age of the Child . . . 13
C.2 Effect for Fathers in Nuclear Families by the Age of the Child . . . 14
C.3 Single Parent Headed Households with Children Under 15 . . . 15
C.4 Extended Family (Not Households Heads or Spouses) in Households with Children Under 15, . . . 16
C.5 Households with Children Under 15 (All Family Members) . . . 17
D.1 Event Study: By Marital Status . . . 18
D.2 Event Study: By Urban Status . . . 19
D.3 Event Study: By High HDI . . . 20
D.4 Event Study: By Age Group for Women . . . 21
D.5 Event Study: By Age Group for Men . . . 22
D.6 Event Study: By Education Group for Women . . . 23
D.7 Event Study: By Education Group, Men . . . 24

List of Tables

B.1 Traditional ENOE (2020Q1) versus ETOE (April 2020) . . . 5
B.2 Traditional ENOE (2020Q3) versus ETOE (July 2020) . . . 6

B.3 ETOE Attrition 7
B.4 Descriptive Statistics for the ETOE, Adults 18-64 8
B.5 Descriptive Statistics, Children 6-16 8
B.6 Household Heads and Spouses with At Least One School-aged Child 5-14 9
B.7 Household Heads and Spouses with At Least Child Under Five 10
B.8 Additional Measures of Time Use for Household Heads and Spouses with At Least One School-aged Child 5-14 11
B.9 Additional Measures of Time Use for Household Heads and Spouses with At Least One Child Under Five 12

A Additional Figures

Figure A.1: Questionnaires for Time Use

XI. OTRAS ACTIVIDADES

11. Durante la semana pasada ¿cuánto tiempo dedicó ... a

(Lee las opciones, circula las indicadas, pregunta y anota el tiempo)

98 en horas y 00 en minutos. Realizó la actividad, pero no sabe cuánto tiempo le dedicó.

99 en horas y 00 en minutos. No sabe si realizó la actividad.

	Horas		Minutos
1 estudiar o tomar cursos de capacitación? *(incluye el tiempo dedicado a realizar trabajos escolares)*	__|__	y	__|__
2 cuidar o atender sin pago, *de manera exclusiva* a niños, ancianos, enfermos o discapacitados? *(bañarlos, cambiarlos)*	__|__	y	__|__
3 realizar compras, llevar cuentas o realizar trámites para el hogar o encargarse de la seguridad? *(como guardar el automóvil)*	__|__	y	__|__
4 llevar a algún miembro del hogar a la escuela, cita médica u otra actividad?	__|__	y	__|__
5 construir o ampliar su vivienda?	__|__	y	__|__
6 reparar o dar mantenimiento a su vivienda, muebles, aparatos electrodomésticos o vehículos?	__|__	y	__|__
7 realizar los quehaceres de su hogar? *(lavar, planchar, preparar y servir alimentos, barrer)*	__|__	y	__|__
8 prestar servicios gratuitos a su comunidad? *(conseguir despensas, cuidar personas en un hospital)*	__|__	y	__|__

0 Exclusivo capturista

TERMINA

XI. OTHER ACTIVITIES

11. During the past week, how long dedicated ... to

(Read the options, circle the indicated ones, ask and write down the time)

98 in hours and 00 in minutes . You did the activity, but you don't know how much time devoted to it.

99 in hours and 00 in minutes. You do not know if you did the activity.

	Hours		Minutes
**1 study or take courses training?** *(includes time dedicated to doing jobs schoolchildren)*		and	
**2 care or attend without payment** ***of exclusively*** **for children, elderly, sick or disabled?** *(bathing, change them)*		and	
**3 make purchases, keep accounts or carry out procedures for the home or take care of security?** *(how to store the car)*		and	
**4 bring a member of the household to school, doctor's appointment or other exercise?**		and	
**5 build or expand your home?**		and	
**6 repair or maintain your home, furniture, appliances appliances or vehicles?**		and	
**7 do your chores home?** *(to wash, iron, prepare and serve food, sweep)*		and	
**8 provide free services to your community?** *(to get pantries, take care of people in a hospital)*		and	

NOTES: ENOE documentation. Note that the English version is translated through Google Translate.

Figure A.2: Alternative Specifications for the Grouped Post-period Results

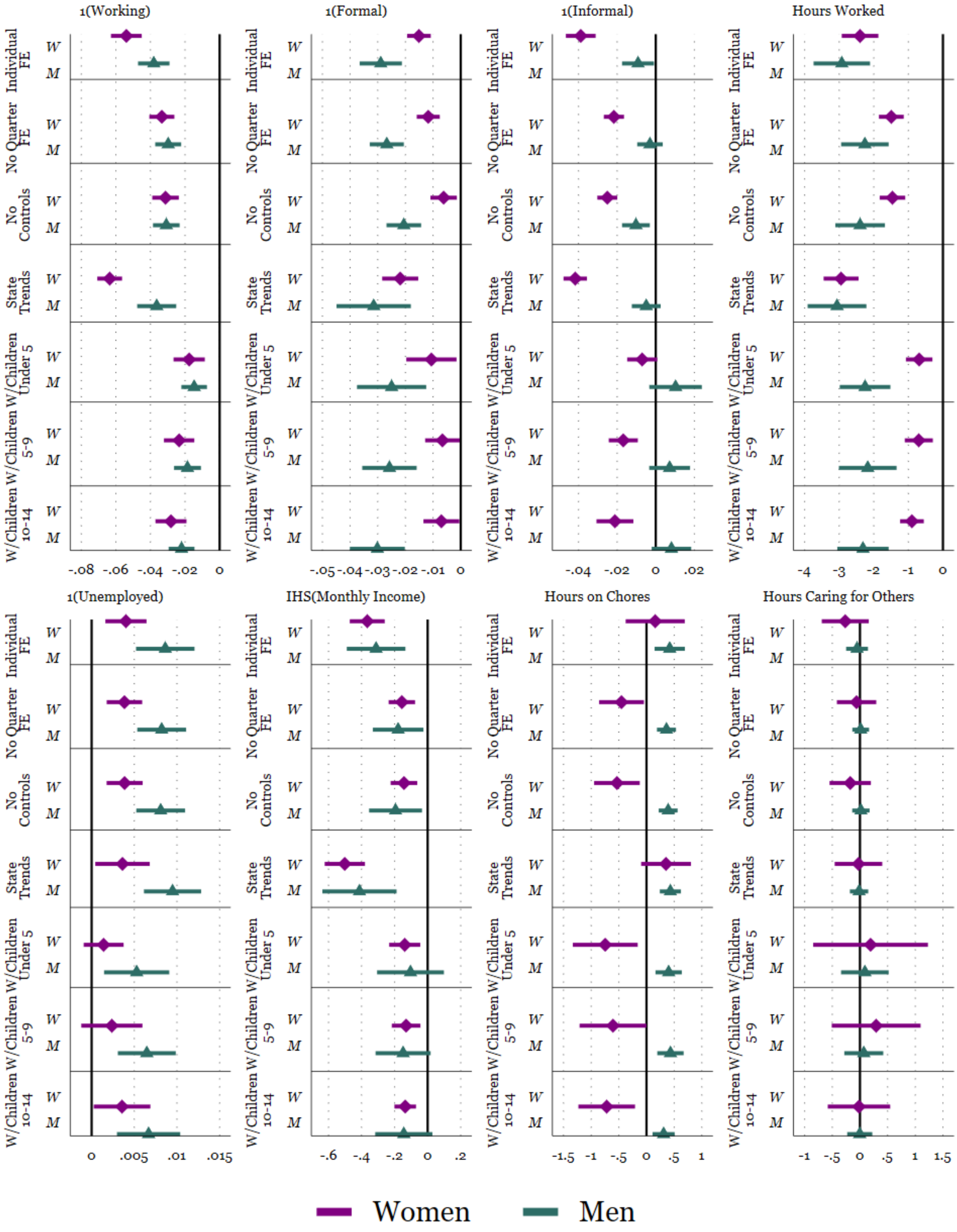

NOTES: OLS coefficients reported from Equation 3. The coefficient indicates the COVID-19 pandemic recession, which equals one for periods 2020Q3 and after. Baseline fixed effects include quarter fixed effects and state-level fixed effects. Controls include indicators for the individual's education and the individual's age. Sample weights are applied using the ENOE's specified sample weights. Each sample is men and women 18-64 from the ENOE, with several modifications. The first change is adding individual fixed effects. The second change removes quarterly fixed effects. The third change is removing controls. Then, fourth, we add state-level trends. Then, we subset to household heads and spouses by the presence of children–under 5, 5-9, and 10-14. All samples use the ENOE and Equation 3, with the noted modification. Robust standard errors are clustered at the state level and are reported in parentheses. ***, **, * represent statistical significance at 1, 5 and 10 percent levels. Individual-level data from the traditional face-to-face National Occupation and Employment Survey (ENOE) 2019Q1-2020Q1 and 2020Q3-2021Q2.

B Additional Tables

Table B.1: Traditional ENOE (2020Q1) versus ETOE (April 2020)

	ENOE 2020Q1		ETOE 2020 April	
	Mean	St. Dev.	Mean	St. Dev.
**Employment**				
1(Working)	0.473	0.499	0.368	0.482
Hours Worked	19.332	23.954	10.797	19.927
1(Unemployed)	0.017	0.129	0.018	0.133
**Sector**				
1(Formal)	0.206	0.405	0.191	0.393
1(Informal)	0.267	0.442	0.176	0.381
1(Construction/Manufac.)	0.114	0.318	0.082	0.274
1(Trade)	0.092	0.290	0.061	0.240
1(Service)	0.203	0.402	0.173	0.378
1(Agriculture)	0.057	0.232	0.046	0.210
1(Other)	0.006	0.078	0.006	0.074
**Time Use**				
Hours on House	15.619	13.350	15.504	12.155
Hours on Chores	12.984	12.050	13.089	11.420
Hours on House Maintenance	0.208	1.286	0.696	3.183
Hours Home Purchasing	1.957	2.495	1.561	2.075
Hours on Building	0.027	0.760	0.078	1.512
Hours Caring for Others	4.264	9.940	5.230	12.273
Hours on Schoolwork	6.265	14.329	2.104	7.096
Hours Transporting	0.443	1.577	0.080	0.762
Hours on Community	0.076	1.216	0.057	0.793
**Income**				
Monthly Income	2,247.638	4,905.872	1,847.330	4,728.995
**Characteristics**				
Age	35.270	20.518	35.628	20.691
1(Education-Less than Primary)	0.088	0.283	0.080	0.271
1(Education-Primary)	0.298	0.457	0.319	0.466
1(Education-Middle)	0.258	0.438	0.257	0.437
1(Education-High School)	0.173	0.378	0.166	0.372
1(Education-Above High School)	0.184	0.387	0.179	0.383
N	381,059		27,419	

NOTES: The first two columns are from the National Occupation and Employment Survey (ENOE). The second two columns are from Encuesta Telefónica de Ocupación y Empleo (ETOE) 2020. All individuals in the specified waves included in the sample. Sample weights are applied using the ENOE's specified sample weights.

Table B.2: Traditional ENOE (2020Q3) versus ETOE (July 2020)

	ENOE 2020Q3		ETOE 2020 July	
	Mean	St. Dev.	Mean	St. Dev.
**Employment**				
1(Working)	0.437	0.496	0.404	0.491
Hours Worked	17.167	23.197	14.809	22.226
1(Unemployed)	0.022	0.147	0.026	0.159
**Sector**				
1(Formal)	0.188	0.391	0.206	0.404
1(Informal)	0.249	0.432	0.198	0.398
1(Construction/Manufac.)	0.110	0.313	0.081	0.273
1(Trade)	0.084	0.277	0.088	0.283
1(Service)	0.173	0.378	0.202	0.402
1(Agriculture)	0.064	0.246	0.029	0.168
1(Other)	0.006	0.076	0.004	0.063
**Time Use**				
Hours on House	15.806	13.188	15.378	12.021
Hours on Chores	13.564	12.393	13.392	11.346
Hours on House Maintenance	0.317	1.797	0.288	1.803
Hours Home Purchasing	1.809	2.534	1.654	2.089
Hours on Building	0.029	0.788	0.008	0.350
Hours Caring for Others	4.192	9.968	3.935	10.024
Hours on Schoolwork	2.619	8.472	1.058	4.857
Hours Transporting	0.087	0.906	0.036	0.493
Hours on Community	0.066	1.209	0.023	0.472
**Income**				
Monthly Income	1,974.996	4,575.980	1,657.288	4,137.574
**Characteristics**				
Age	35.268	20.556	35.361	20.240
1(Education-Less than Primary)	0.085	0.280	0.070	0.255
1(Education-Primary)	0.303	0.459	0.273	0.445
1(Education-Middle)	0.258	0.438	0.236	0.425
1(Education-High School)	0.173	0.378	0.195	0.396
1(Education-Above High School)	0.181	0.385	0.226	0.418
N	219,974		18,781	

NOTES: The first two columns are from the National Occupation and Employment Survey (ENOE). The second two columns are from Encuesta Telefónica de Ocupación y Empleo (ETOE) 2020. All individuals in the specified waves included in the sample. Sample weights are applied using the ENOE's specified sample weights.

Table B.3: ETOE Attrition

	Leave Sample				
	(1)	(2)	(3)	(4)	(5)
1(Education-Primary)	0.044	0.048	0.056	0.062	0.060
	(0.109)	(0.110)	(0.109)	(0.108)	(0.107)
1(Education-Middle)	0.035	0.035	0.047	0.057	0.055
	(0.101)	(0.101)	(0.101)	(0.097)	(0.097)
1(Education-High School)	0.045	0.041	0.052	0.063	0.061
	(0.101)	(0.102)	(0.102)	(0.098)	(0.097)
1(Education-Above High School)	0.000	-0.001	0.010	0.025	0.023
	(0.099)	(0.099)	(0.098)	(0.095)	(0.093)
Age		-0.001	0.001	0.001	0.001
		(0.001)	(0.002)	(0.001)	(0.001)
1(Married)			-0.005	-0.008	-0.009
			(0.061)	(0.062)	(0.061)
1(Cohabitating)			0.072	0.072	0.070
			(0.069)	(0.070)	(0.070)
1(Divorced/Separated)			-0.010	-0.007	-0.009
			(0.059)	(0.059)	(0.058)
1(Single)			0.070	0.081	0.079
			(0.077)	(0.079)	(0.078)
Number Children				0.013	0.013
				(0.010)	(0.010)
1(Working)					0.008
					(0.014)
Observations	18,300	18,300	18,300	18,300	18,300
Adjusted R-squared	0.09	0.09	0.09	0.10	0.10
State Fixed Effects	X	X	X	X	X

NOTES: The analysis considers the probability of leaving the ETOE early (attrition) based on individual demographic characteristics. The sample includes the first month of the ETOE survey. The sample is also based on the analysis sample, men and women 18-64. Fixed effects included for the state. Omitted group for education includes the less than a primary level of education (“ninguno” and “preescolar”). Primary includes “primaria.” Middle includes “secundaria.” High school captures “preparatoria o bachillerato.” Above high school includes “carrera técnica” or technical education, “profesional” or professional, “maestría” or masters, and “doctorado” or doctorate. The omitted group for marital status is widowed. Sample weights are applied using the ENOE’s specified sample weights. Robust standard errors are clustered at the state level and are reported in parentheses. ***, **, * represent statistical significance at 1, 5 and 10 percent levels. Encuesta Telefónica de Ocupación y Empleo (ETOE) 2020.

Table B.4: Descriptive Statistics for the ETOE, Adults 18-64

	Women Pre		Women Post		Men Pre		Men Post	
	Mean	St. Dev.	Mean	St. Dev.	Mean	St. Dev.	Mean	St. Dev.
**Employment**								
1(Working)	0.576	0.494	0.426	0.494	0.832	0.373	0.704	0.457
Hours Worked	21.256	22.658	12.875	19.918	37.889	23.468	26.297	24.628
1(Unemployed)	0.025	0.155	0.019	0.135	0.037	0.189	0.043	0.203
**Sector**								
1(Formal)	0.281	0.449	0.226	0.418	0.456	0.498	0.363	0.481
1(Informal)	0.295	0.456	0.200	0.400	0.376	0.484	0.341	0.474
1(Construction/Manufac.)	0.082	0.274	0.070	0.254	0.238	0.426	0.191	0.393
1(Trade)	0.133	0.340	0.091	0.287	0.133	0.340	0.113	0.317
1(Service)	0.345	0.475	0.252	0.434	0.382	0.486	0.283	0.451
1(Agriculture)	0.011	0.106	0.012	0.107	0.064	0.245	0.106	0.308
1(Other)	0.005	0.068	0.002	0.047	0.015	0.121	0.011	0.102
**Time Use**								
Hours on House	23.565	14.131	22.804	12.326	7.874	6.907	8.738	7.491
Hours on Chores	19.881	12.628	20.429	11.659	5.402	5.502	6.101	6.090
Hours on House Maintenance	0.038	0.524	0.093	1.197	0.533	2.338	0.905	3.040
Hours Home Purchasing	2.764	2.648	2.196	2.566	1.530	1.885	1.553	1.908
Hours on Building	0.015	0.824	0.003	0.227	0.060	1.154	0.095	1.537
Hours Caring for Others	7.550	13.031	8.165	14.370	2.314	6.110	2.233	6.364
Hours on Schoolwork	2.838	10.385	1.467	6.519	3.464	11.356	1.946	7.485
Hours Transporting	0.868	2.173	0.083	0.794	0.349	1.293	0.083	0.742
Hours on Community	0.071	0.940	0.043	0.761	0.080	0.956	0.063	0.751
**Income**								
Monthly Income	2,642.430	4,970.825	1,849.458	4,404.384	4,898.869	6,678.923	3,534.942	5,836.540
**Characteristics**								
Age	39.118	13.022	38.715	12.882	38.590	13.229	38.890	13.250
1(Education-Less than Primary)	0.016	0.125	0.022	0.146	0.017	0.128	0.021	0.143
1(Education-Primary)	0.151	0.358	0.196	0.397	0.141	0.348	0.181	0.385
1(Education-Middle)	0.266	0.442	0.274	0.446	0.270	0.444	0.280	0.449
1(Education-High School)	0.246	0.431	0.231	0.421	0.273	0.446	0.258	0.438
1(Education-Above High School)	0.322	0.467	0.277	0.447	0.299	0.458	0.260	0.439
N	34,516		55,045		31,652		50,866	

SOURCE: Data includes National Occupation and Employment Survey (ENOE) 2019-2020Q1. For the post-period, April-November 2020, data is from the Encuesta Telefónica de Ocupación y Empleo (ETOE) 2020. Sample weights are applied using the ENOE's specified sample weights.

Table B.5: Descriptive Statistics, Children 6-16

	Girls Pre		Girls Post		Boys Pre		Boys Post	
	Mean	St. Dev.	Mean	St. Dev.	Mean	St. Dev.	Mean	St. Dev.
1(In School)	0.947	0.224	0.942	0.233	0.938	0.241	0.932	0.253
Hours on Schoolwork	32.341	14.594	24.258	13.913	32.250	14.039	23.477	13.341
1(Working)	0.030	0.171	0.032	0.175	0.072	0.259	0.080	0.271
Hours Worked	0.747	5.250	0.858	5.782	2.127	9.094	2.552	10.052
Hours on House	8.183	6.874	8.843	7.398	5.004	4.839	5.383	5.288
Hours Caring for Others	1.105	4.649	1.336	5.262	0.458	2.550	0.524	2.920
N	186,742		111,580		195,527		116,867	

NOTES: The sample of children includes individuals who are 6 to 16. Sample weights are applied using the ENOE's specified sample weights. Individual-level data from the traditional face-to-face National Occupation and Employment Survey (ENOE) 2019Q1-2020Q1 and 2020Q3-2021Q2.

Table B.6: Household Heads and Spouses with At Least One School-aged Child 5-14

Panel A: Mothers

	1(Work -ing)	Hours Worked	1(For- mal)	Formal Hours	1(Infor- mal)	Informal Hours	Hours Chores	Hours Caring	IHS Income
	(1)	(2)	(3)	(4)	(5)	(6)	(7)	(8)	(9)
1(COVID-19 Pandemic)	-0.025***	-0.823***	-0.007**	-0.668**	-0.018***	0.734**	-0.642**	0.119	-0.135***
	(0.003)	(0.140)	(0.003)	(0.294)	(0.003)	(0.307)	(0.263)	(0.338)	(0.031)
Observations	232,047	232,047	232,047	51,050	232,047	58,568	229,259	229,259	232,047
Adjusted R-squared	0.068	0.041	0.189	0.107	0.031	0.019	0.067	0.132	0.050
Pre-Mean Dependent	0.438	14.939	0.177	39.693	0.261	30.253	25.667	12.147	2.874
Post-Mean Dependent	0.415	14.204	0.173	38.788	0.242	30.928	25.088	12.212	2.777
Quarter FE	X	X	X	X	X	X	X	X	X
State FE	X	X	X	X	X	X	X	X	X
Controls	X	X	X	X	X	X	X	X	X

Panel B: Fathers

	1(Work -ing)	Hours Worked	1(For- mal)	Formal Hours	1(Infor- mal)	Informal Hours	Hours Chores	Hours Caring	IHS Income
	(1)	(2)	(3)	(4)	(5)	(6)	(7)	(8)	(9)
1(COVID-19 Pandemic)	-0.020***	-2.098***	-0.027***	-1.164***	0.007	-1.204***	0.360***	0.043	-0.131
	(0.003)	(0.362)	(0.004)	(0.363)	(0.004)	(0.290)	(0.097)	(0.141)	(0.080)
Observations	232,051	232,051	232,051	120,748	232,051	97,913	181,812	181,812	232,051
Adjusted R-squared	0.021	0.028	0.221	0.046	0.205	0.032	0.031	0.082	0.088
Pre-Mean Dependent	0.949	44.037	0.459	47.953	0.490	44.937	4.172	3.836	6.586
Post-Mean Dependent	0.928	41.756	0.435	46.666	0.493	43.482	4.505	3.906	6.506
Quarter FE	X	X	X	X	X	X	X	X	X
State FE	X	X	X	X	X	X	X	X	X
Controls	X	X	X	X	X	X	X	X	X

NOTES: OLS coefficients reported from Equation 3. The coefficient indicates the COVID-19 pandemic recession, which equals one for periods 2020Q3 and after. Baseline fixed effects include quarter fixed effects and state-level fixed effects. Controls include indicators for the individual's education and the individual's age. Sample weights are applied using the ENOE's specified sample weights. The main sample includes individuals who are 18 to 64. We also subset to household heads and their spouses with children 5-14 present in the household. Robust standard errors are clustered at the state level and are reported in parentheses. ***, **, * represent statistical significance at 1, 5 and 10 percent levels. Individual-level data from the traditional face-to-face National Occupation and Employment Survey (ENOE) 2019Q1-2020Q1 and 2020Q3-2021Q2.

Table B.7: Household Heads and Spouses with At Least Child Under Five

Panel A: Mothers

	1(Working)	Hours Worked	1(Formal)	Formal Hours	1(Informal)	Informal Hours	Hours Chores	Hours Caring	IHS Income
	(1)	(2)	(3)	(4)	(5)	(6)	(7)	(8)	(9)
1(COVID-19 Pandemic)	-0.018***	-0.687***	-0.011**	-1.157***	-0.007*	0.826**	-0.746**	0.191	-0.139***
	(0.004)	(0.188)	(0.004)	(0.379)	(0.004)	(0.353)	(0.287)	(0.509)	(0.046)
Observations	112,896	112,896	112,896	22,830	112,896	23,574	111,687	111,687	112,896
Adjusted R-squared	0.086	0.054	0.192	0.089	0.018	0.029	0.076	0.074	0.063
Pre-Mean Dependent	0.373	12.473	0.163	38.837	0.210	29.283	24.551	20.602	2.543
Post-Mean Dependent	0.358	11.889	0.154	37.475	0.203	30.044	23.888	20.740	2.446
Quarter FE	X	X	X	X	X	X	X	X	X
State FE	X	X	X	X	X	X	X	X	X
Controls	X	X	X	X	X	X	X	X	X

Panel B: Fathers

	1(Working)	Hours Worked	1(Formal)	Formal Hours	1(Informal)	Informal Hours	Hours Chores	Hours Caring	IHS Income
	(1)	(2)	(3)	(4)	(5)	(6)	(7)	(8)	(9)
1(COVID-19 Pandemic)	-0.015***	-2.252***	-0.025***	-1.447***	0.010	-1.611***	0.400***	0.089	-0.104
	(0.004)	(0.359)	(0.006)	(0.331)	(0.007)	(0.350)	(0.117)	(0.211)	(0.099)
Observations	112,896	112,896	112,896	59,948	112,896	47,535	93,751	93,751	112,896
Adjusted R-squared	0.011	0.021	0.212	0.046	0.197	0.032	0.033	0.072	0.089
Pre-Mean Dependent	0.957	44.804	0.468	48.226	0.489	45.513	4.165	6.453	7.034
Post-Mean Dependent	0.941	42.381	0.449	46.605	0.492	43.592	4.561	6.608	6.964
Quarter FE	X	X	X	X	X	X	X	X	X
State FE	X	X	X	X	X	X	X	X	X
Controls	X	X	X	X	X	X	X	X	X

NOTES: OLS coefficients reported from Equation 3. The coefficient indicates the COVID-19 pandemic recession, which equals one for periods 2020Q3 and after. Baseline fixed effects include quarter fixed effects and state-level fixed effects. Controls include indicators for the individual's education and the individual's age. Sample weights are applied using the ENOE's specified sample weights. The main sample includes individuals who are 18 to 64. We also subset to household heads and their spouses with one child under five present in the household. Robust standard errors are clustered at the state level and are reported in parentheses. ***, **, * represent statistical significance at 1, 5 and 10 percent levels. Individual-level data from the traditional face-to-face National Occupation and Employment Survey (ENOE) 2019Q1-2020Q1 and 2020Q3-2021Q2.

Table B.8: Additional Measures of Time Use for Household Heads and Spouses with At Least One School-aged Child 5-14

Panel A: Mothers

	Hours House	Hours Maintenance	Hours Purchasing	Hours Building	Hours Transporting	Hours School	Hours Community
	(1)	(2)	(3)	(4)	(5)	(6)	(7)
1(COVID-19 Pandemic)	-2.458***	0.019***	-0.323***	0.004	-1.516***	0.045	-0.026**
	(0.290)	(0.006)	(0.038)	(0.003)	(0.083)	(0.030)	(0.013)
Observations	229,259	229,259	229,259	229,259	229,259	227,635	229,259
Adjusted R-squared	0.069	0.003	0.120	0.000	0.126	0.021	0.001
Pre-Mean Dependent	30.741	0.036	3.284	0.006	1.748	0.274	0.077
Post-Mean Dependent	28.255	0.057	2.896	0.010	0.205	0.322	0.052
Quarter FE	X	X	X	X	X	X	X
State FE	X	X	X	X	X	X	X
Controls	X	X	X	X	X	X	X

Panel B: Fathers

	Hours House	Hours Maintenance	Hours Purchasing	Hours Building	Hours Transporting	Hours School	Hours Community
	(1)	(2)	(3)	(4)	(5)	(6)	(7)
1(COVID-19 Pandemic)	0.080	0.134***	0.009	0.026*	-0.449***	0.071***	-0.014
	(0.141)	(0.043)	(0.050)	(0.013)	(0.033)	(0.024)	(0.023)
Observations	181,812	181,812	181,812	181,812	181,812	180,774	181,812
Adjusted R-squared	0.027	0.021	0.042	0.004	0.046	0.025	0.027
Pre-Mean Dependent	7.303	0.680	1.782	0.086	0.582	0.212	0.118
Post-Mean Dependent	7.369	0.823	1.790	0.115	0.136	0.284	0.112
Quarter FE	X	X	X	X	X	X	X
State FE	X	X	X	X	X	X	X
Controls	X	X	X	X	X	X	X

NOTES: OLS coefficients reported from Equation 3. The coefficient indicates the COVID-19 pandemic recession, which equals one for periods 2020Q3 and after. Baseline fixed effects include quarter fixed effects and state-level fixed effects. Controls include indicators for the individual's education and the individual's age. Sample weights are applied using the ENOE's specified sample weights. The main sample includes individuals who are 18 to 64. We also subset to household heads and their spouses with one child 5-14 present in the household. Robust standard errors are clustered at the state level and are reported in parentheses. ***, **, * represent statistical significance at 1, 5 and 10 percent levels. Individual-level data from the traditional face-to-face National Occupation and Employment Survey (ENOE) 2019Q1-2020Q1 and 2020Q3-2021Q2.

Table B.9: Additional Measures of Time Use for Household Heads and Spouses with At Least One Child Under Five

Panel A: Mothers

	Hours House	Hours Maintenance	Hours Purchasing	Hours Building	Hours Transporting	Hours School	Hours Community
	(1)	(2)	(3)	(4)	(5)	(6)	(7)
1(COVID-19 Pandemic)	-2.402***	0.020***	-0.357***	0.001	-1.321***	0.013	-0.005
	(0.340)	(0.006)	(0.049)	(0.002)	(0.072)	(0.052)	(0.011)
Observations	111,687	111,687	111,687	111,687	111,687	110,534	111,687
Adjusted R-squared	0.082	0.003	0.100	0.001	0.105	0.024	0.001
Pre-Mean Dependent	29.305	0.030	3.159	0.006	1.559	0.373	0.056
Post-Mean Dependent	26.931	0.051	2.755	0.008	0.230	0.376	0.048
Quarter FE	X	X	X	X	X	X	X
State FE	X	X	X	X	X	X	X
Controls	X	X	X	X	X	X	X

Panel B: Fathers

	Hours House	Hours Maintenance	Hours Purchasing	Hours Building	Hours Transporting	Hours School	Hours Community
	(1)	(2)	(3)	(4)	(5)	(6)	(7)
1(COVID-19 Pandemic)	0.139	0.107**	-0.022	0.017	-0.363***	0.003	0.015
	(0.140)	(0.040)	(0.053)	(0.011)	(0.028)	(0.035)	(0.027)
Observations	93,751	93,751	93,751	93,751	93,751	92,939	93,751
Adjusted R-squared	0.032	0.021	0.041	0.003	0.038	0.027	0.017
Pre-Mean Dependent	7.100	0.550	1.819	0.070	0.495	0.296	0.100
Post-Mean Dependent	7.266	0.670	1.806	0.089	0.139	0.295	0.121
Quarter FE	X	X	X	X	X	X	X
State FE	X	X	X	X	X	X	X
Controls	X	X	X	X	X	X	X

NOTES: OLS coefficients reported from Equation 3. The coefficient indicates the COVID-19 pandemic recession, which equals one for periods 2020Q3 and after. Baseline fixed effects include quarter fixed effects and state-level fixed effects. Controls include indicators for the individual's education and the individual's age. Sample weights are applied using the ENOE's specified sample weights. The main sample includes individuals who are 18 to 64. We also subset to household heads and their spouses with one child under five present in the household. Robust standard errors are clustered at the state level and are reported in parentheses. ***, **, * represent statistical significance at 1, 5 and 10 percent levels. Individual-level data from the traditional face-to-face National Occupation and Employment Survey (ENOE) 2019Q1-2020Q1 and 2020Q3-2021Q2.

C Additional Effects by Households with Children

Figure C.1: Effect for Mothers in Nuclear Families by the Age of the Child

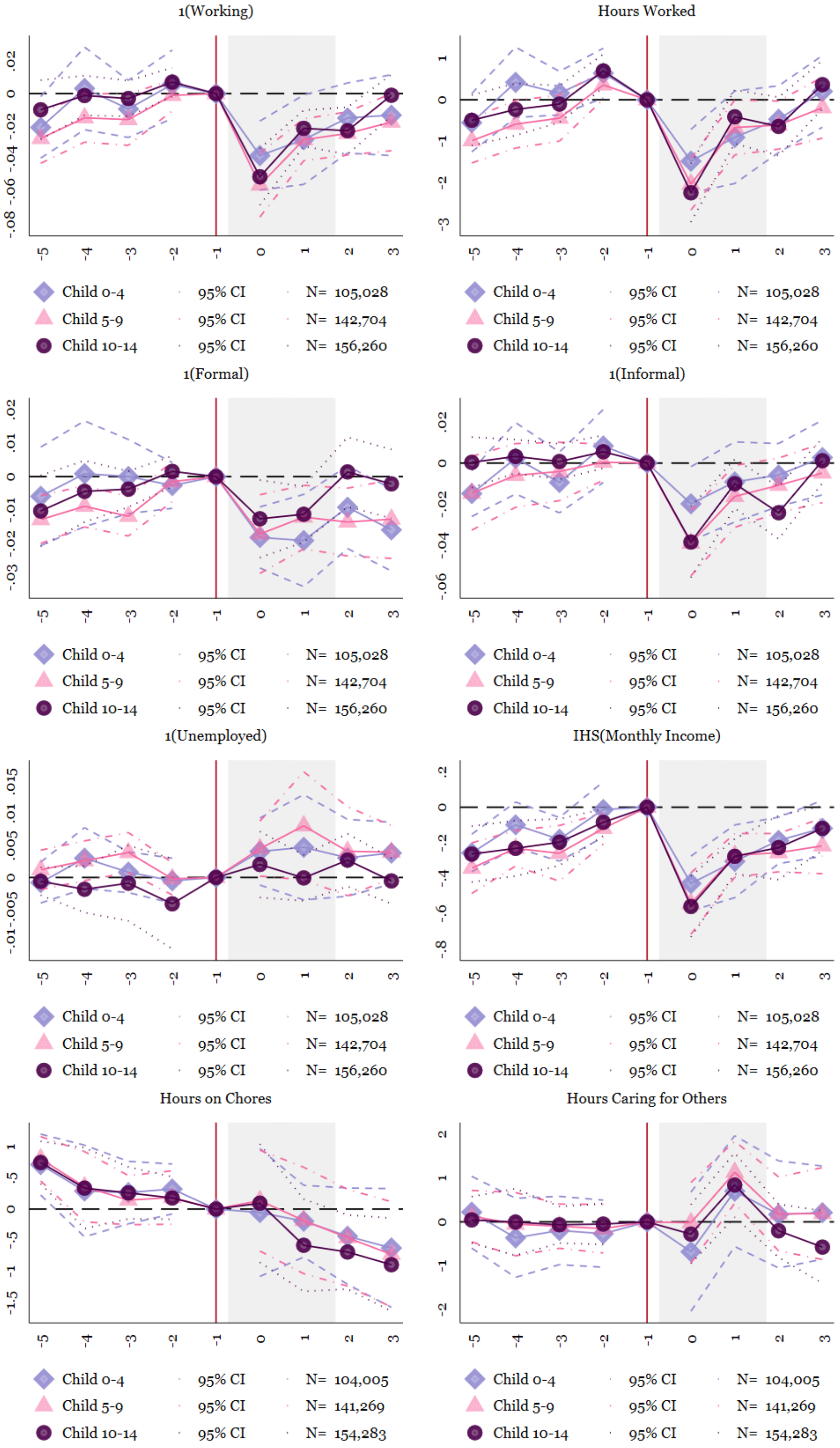

NOTES: OLS coefficients reported from Equation 1. Baseline fixed effects include state-level fixed effects. Controls include indicators for the individual's age and education. Sample weights are applied using the ENOE's specified sample weights. The periods before -1 include 2019Q1-2019Q4. The omitted period represents 2020Q1, represented by the vertical line. There is no 2020Q2, so the post periods represent 2020Q3-2021Q2. The shaded period represents quarters in 2020. The main sample includes individuals who are 18 to 64. We subset to household heads and their spouses with no other family members present except children. The above graph splits this sample into mothers with children under five, from five to nine, and ten to fourteen. Robust standard errors are clustered at the state level. Individual-level data from the traditional face-to-face National Occupation and Employment Survey (ENOE) 2019Q1-2020Q1 and 2020Q3-2021Q2.

Figure C.2: Effect for Fathers in Nuclear Families by the Age of the Child

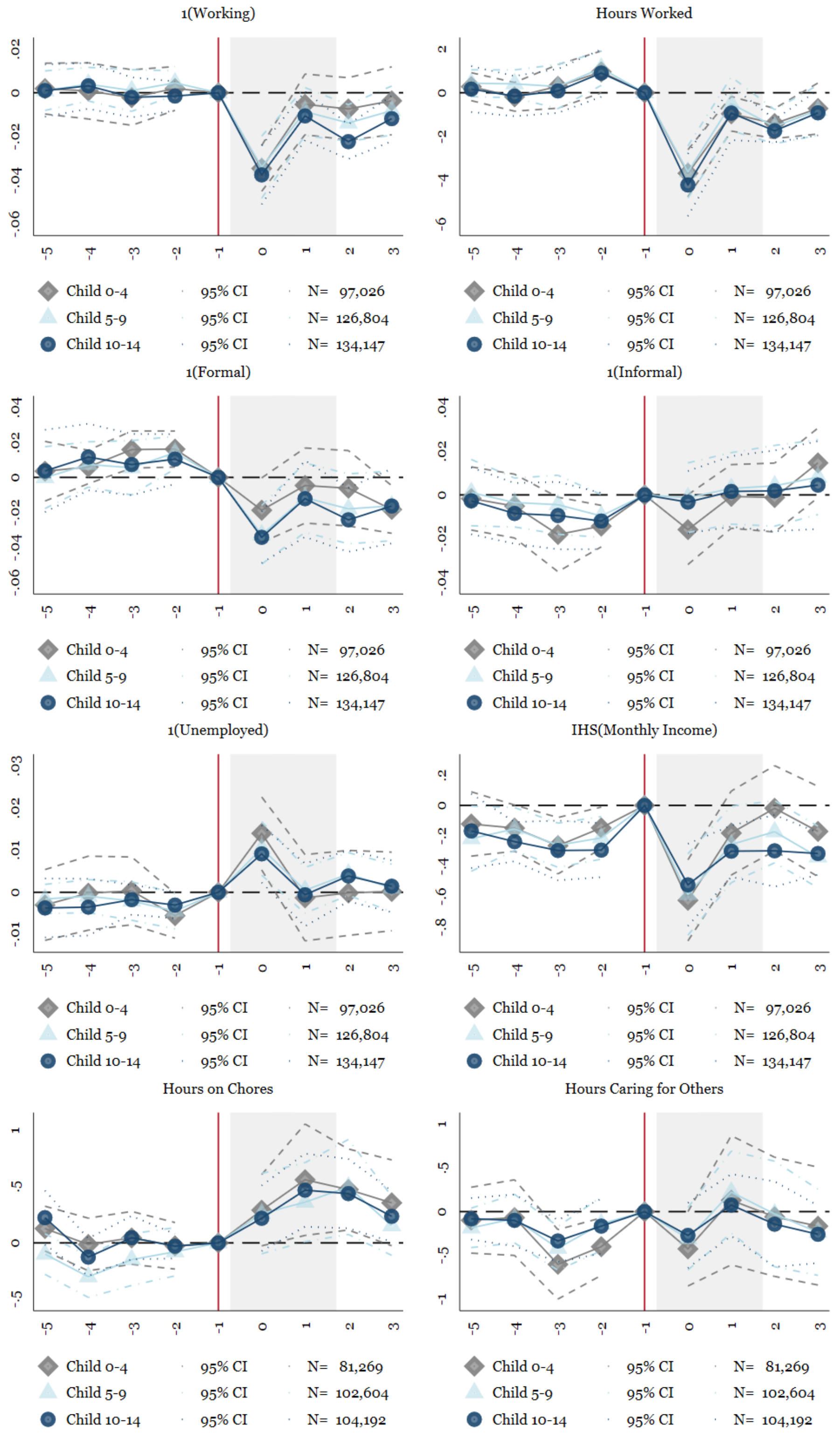

NOTES: OLS coefficients reported from Equation 1. Baseline fixed effects include state-level fixed effects. Controls include indicators for the individual's age and education. Sample weights are applied using the ENOE's specified sample weights. The periods before -1 include 2019Q1-2019Q4. The omitted period represents 2020Q1, represented by the vertical line. There is no 2020Q2, so the post periods represent 2020Q3-2021Q2. The shaded period represents quarters in 2020. The main sample includes individuals who are 18 to 64. We subset to household heads and their spouses with no other family members present except children. The above graph splits this sample into fathers with children under five, from five to nine, and ten to fourteen. Robust standard errors are clustered at the state level. Individual-level data from the traditional face-to-face National Occupation and Employment Survey (ENOE) 2019Q1-2020Q1 and 2020Q3-2021Q2.

Figure C.3: Single Parent Headed Households with Children Under 15

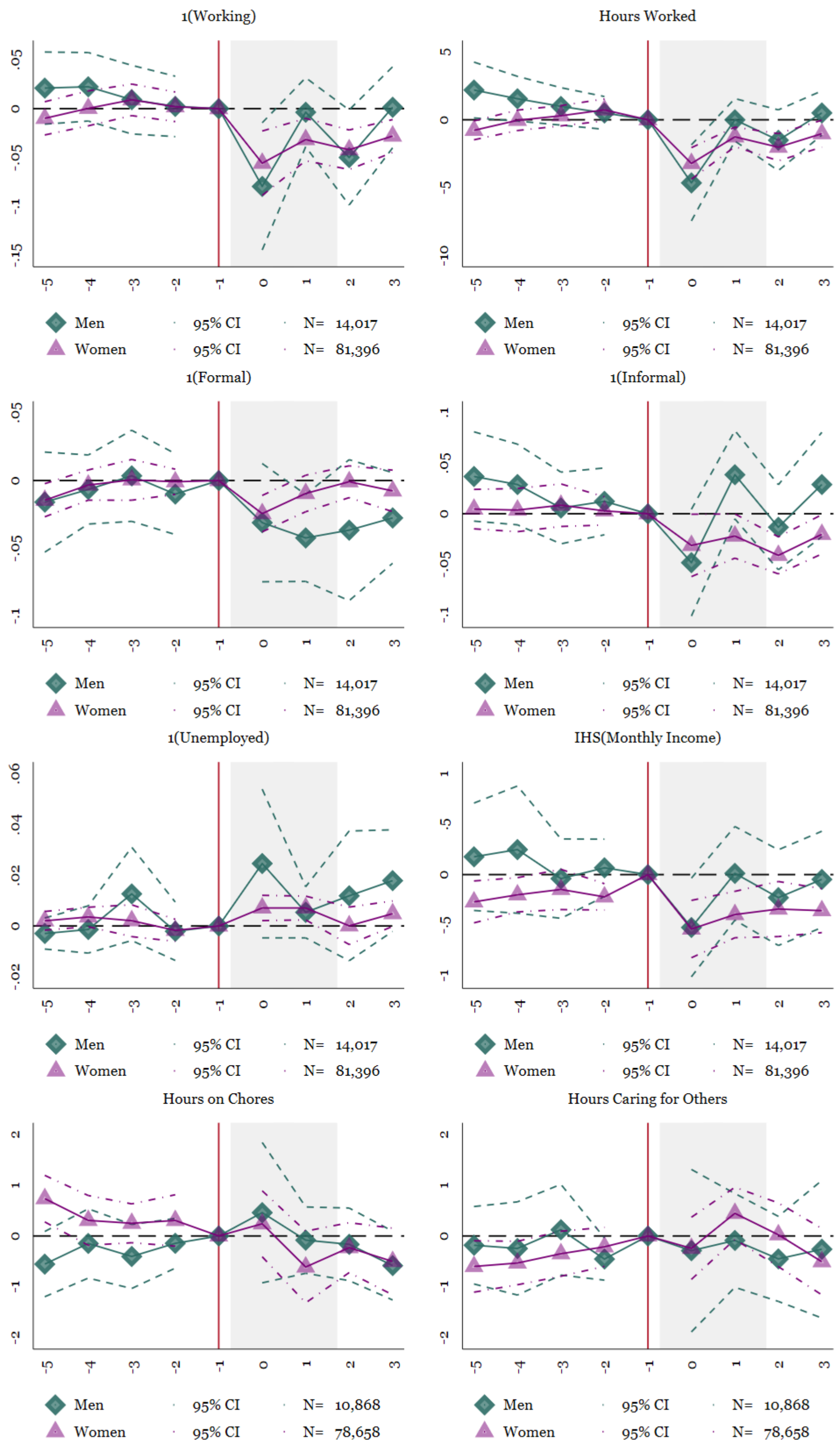

NOTES: OLS coefficients reported from Equation 1. Baseline fixed effects include state-level fixed effects. Controls include indicators for the individual's age and education. Sample weights are applied using the ENOE's specified sample weights. The periods before -1 include 2019Q1-2019Q4. The omitted period represents 2020Q1, represented by the vertical line. There is no 2020Q2, so the post periods represent 2020Q3-2021Q2. The shaded period represents quarters in 2020. The main sample includes individuals who are 18 to 64. We subset to single parent headed households. Robust standard errors are clustered at the state level. Individual-level data from the traditional face-to-face National Occupation and Employment Survey (ENOE) 2019Q1-2020Q1 and 2020Q3-2021Q2.

Figure C.4: Extended Family (Not Households Heads or Spouses) in Households with Children Under 15,

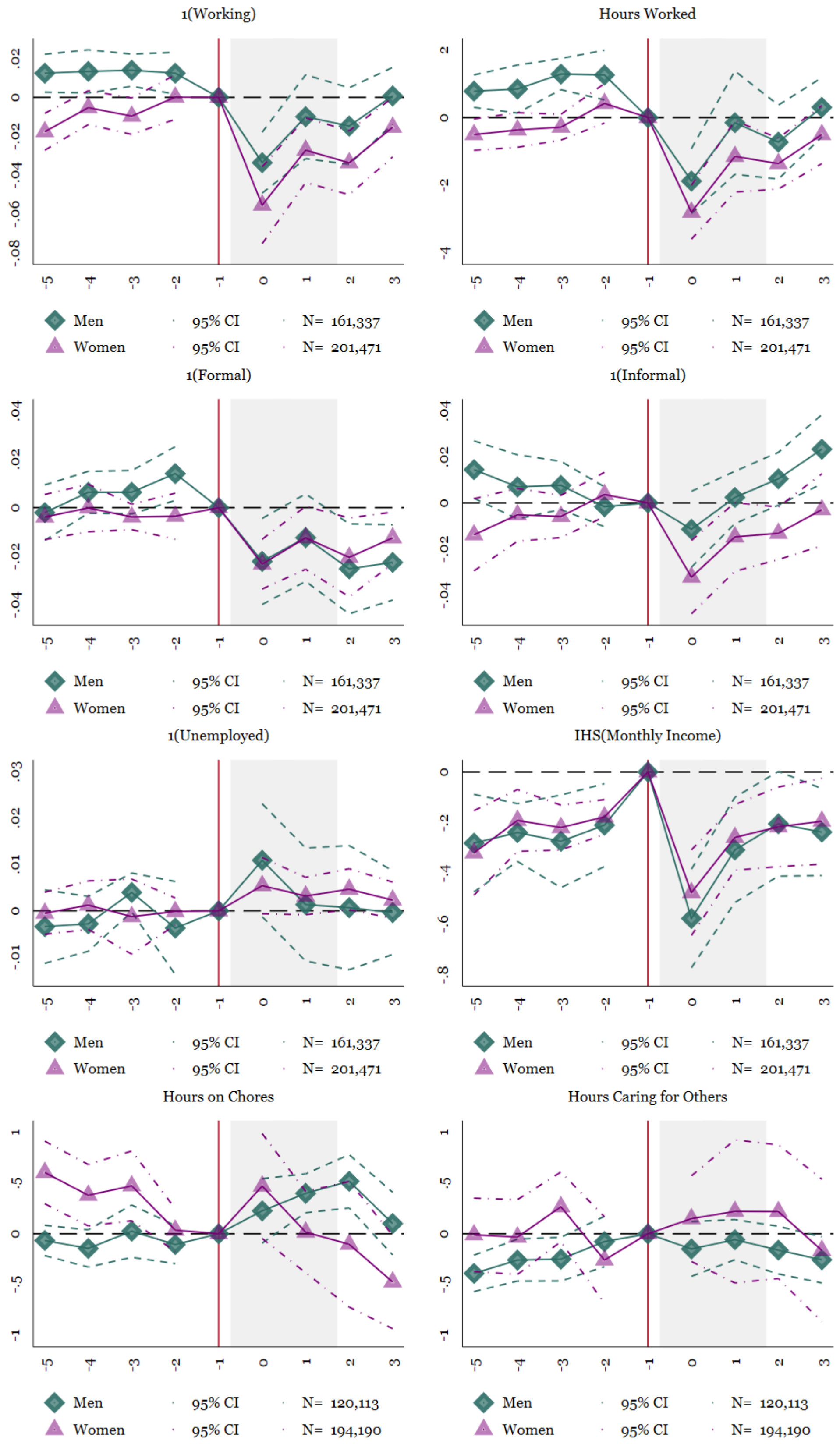

NOTES: OLS coefficients reported from Equation 1. Baseline fixed effects include state-level fixed effects. Controls include indicators for the individual's age and education. Sample weights are applied using the ENOE's specified sample weights. The periods before -1 include 2019Q1-2019Q4. The omitted period represents 2020Q1, represented by the vertical line. There is no 2020Q2, so the post periods represent 2020Q3-2021Q2. The shaded period represents quarters in 2020. The main sample includes individuals who are 18 to 64. We subset to households with children under 15, and consider non-household heads or spouses. Robust standard errors are clustered at the state level. Individual-level data from the traditional face-to-face National Occupation and Employment Survey (ENOE) 2019Q1-2020Q1 and 2020Q3-2021Q2.

Figure C.5: Households with Children Under 15 (All Family Members)

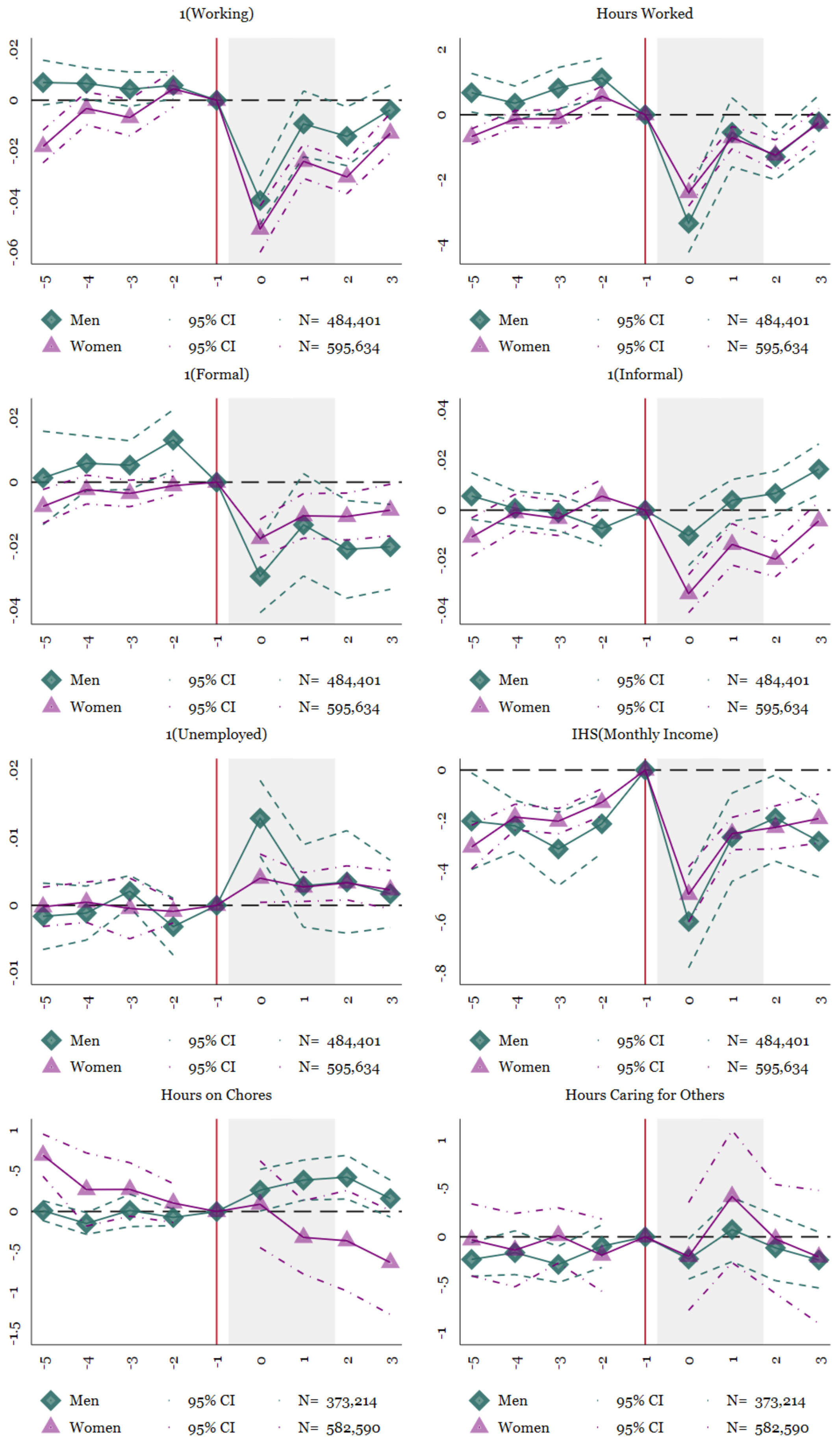

NOTES: OLS coefficients reported from Equation 1. Baseline fixed effects include state-level fixed effects. Controls include indicators for the individual's age and education. Sample weights are applied using the ENOE's specified sample weights. The periods before -1 include 2019Q1-2019Q4. The omitted period represents 2020Q1, represented by the vertical line. There is no 2020Q2, so the post periods represent 2020Q3-2021Q2. The shaded period represents quarters in 2020. The main sample includes individuals who are 18 to 64. We subset to households with children under 15, and consider any individuals present in the household. Robust standard errors are clustered at the state level. Individual-level data from the traditional face-to-face National Occupation and Employment Survey (ENOE) 2019Q1-2020Q1 and 2020Q3-2021Q2.

D Additional Heterogeneous Effects

Figure D.1: Event Study: By Marital Status

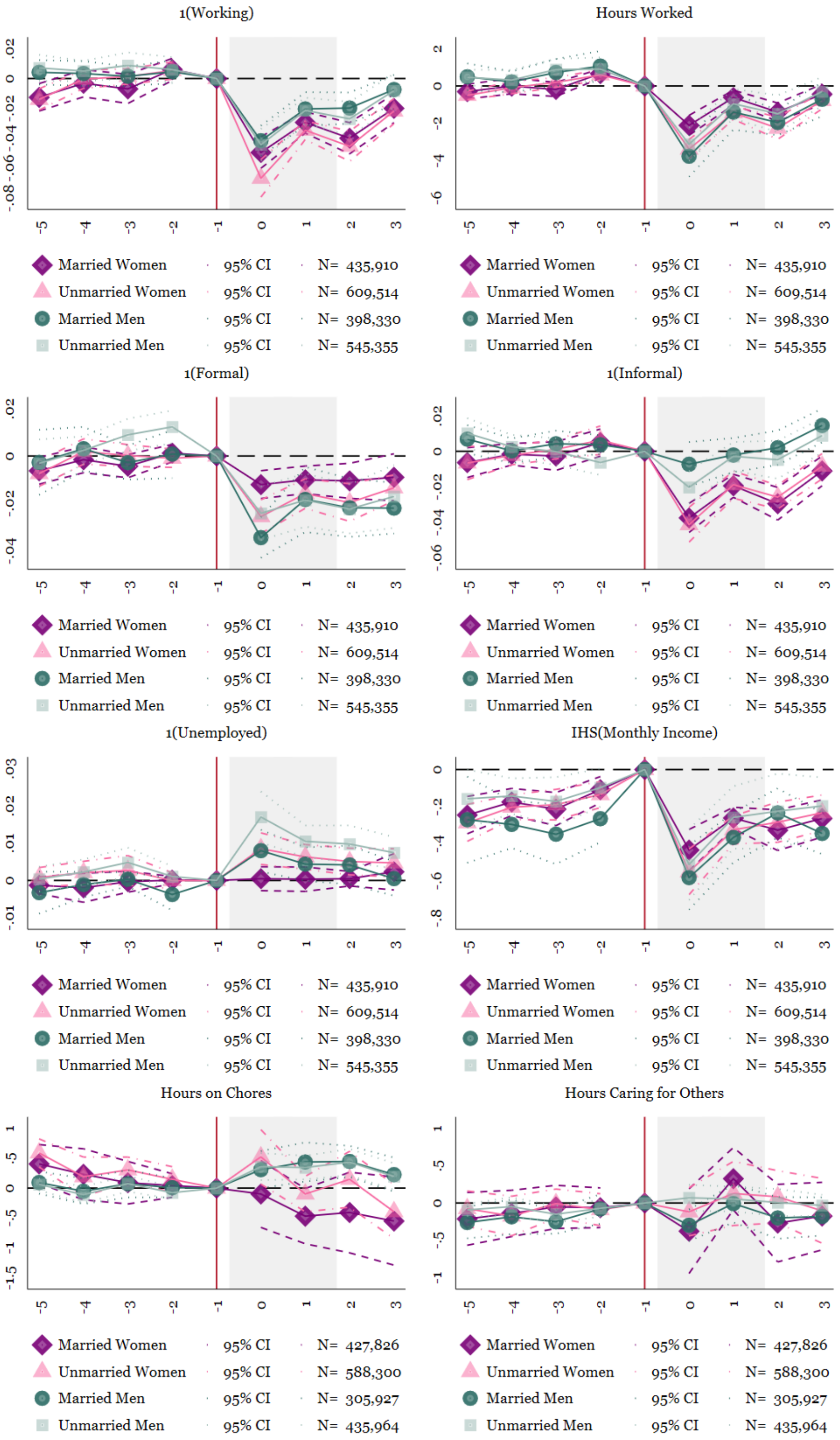

NOTES: OLS coefficients reported from Equation 1. Baseline fixed effects include state-level fixed effects. Controls include indicators for the individual's age and education. Sample weights are applied using the ENOE's specified sample weights. The periods before -1 include 2019Q1-2019Q4. The omitted period represents 2020Q1, represented by the vertical line. There is no 2020Q2, so the post periods represent 2020Q3-2021Q2. The shaded period represents quarters in 2020. The main sample includes individuals who are 18 to 64. Robust standard errors are clustered at the state level. Individual-level data from the traditional face-to-face National Occupation and Employment Survey (ENOE) 2019Q1-2020Q1 and 2020Q3-2021Q2.

Figure D.2: Event Study: By Urban Status

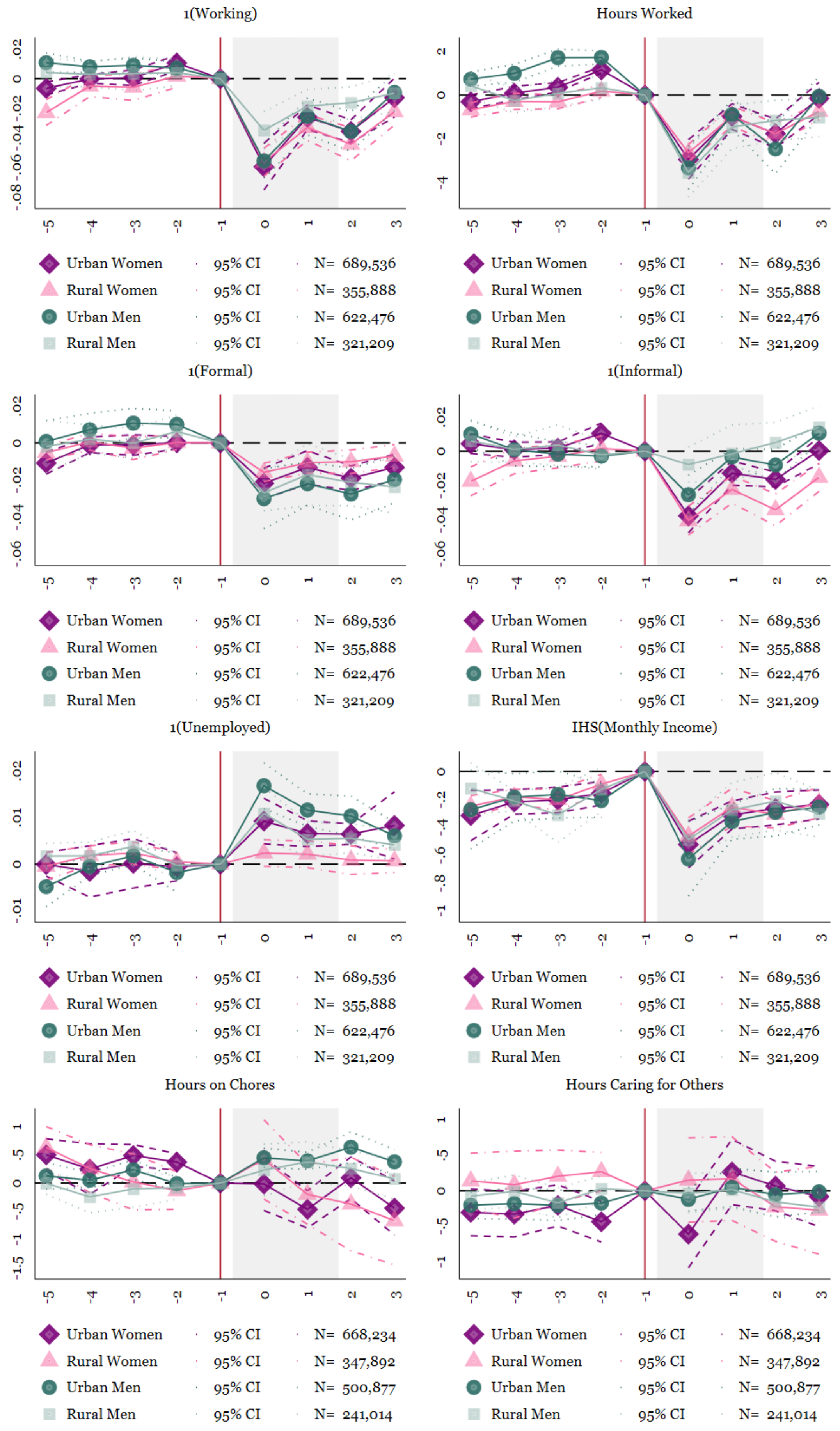

NOTES: OLS coefficients reported from Equation 1. Baseline fixed effects include state-level fixed effects. Controls include indicators for the individual's age and education. Sample weights are applied using the ENOE's specified sample weights. The periods before -1 include 2019Q1-2019Q4. The omitted period represents 2020Q1, represented by the vertical line. There is no 2020Q2, so the post periods represent 2020Q3-2021Q2. The shaded period represents quarters in 2020. The main sample includes individuals who are 18 to 64. Robust standard errors are clustered at the state level. Individual-level data from the traditional face-to-face National Occupation and Employment Survey (ENOE) 2019Q1-2020Q1 and 2020Q3-2021Q2.

Figure D.3: Event Study: By High HDI

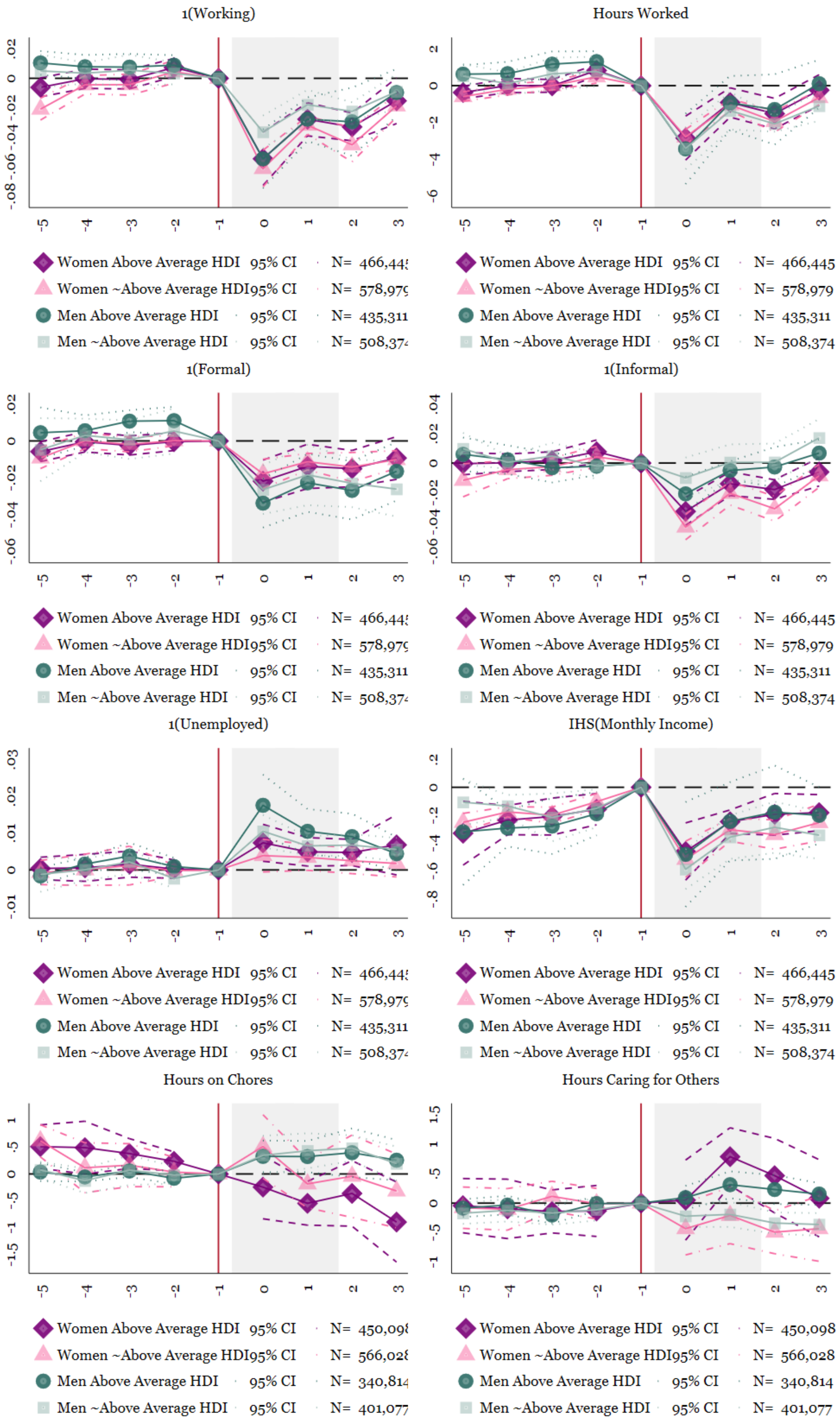

NOTES: OLS coefficients reported from Equation 1. Baseline fixed effects include state-level fixed effects. Controls include indicators for the individual's age and education. Sample weights are applied using the ENOE's specified sample weights. The periods before -1 include 2019Q1-2019Q4. The omitted period represents 2020Q1, represented by the vertical line. There is no 2020Q2, so the post periods represent 2020Q3-2021Q2. The shaded period represents quarters in 2020. The main sample includes individuals who are 18 to 64. Robust standard errors are clustered at the state level. Individual-level data from the traditional face-to-face National Occupation and Employment Survey (ENOE) 2019Q1-2020Q1 and 2020Q3-2021Q2.

Figure D.4: Event Study: By Age Group for Women

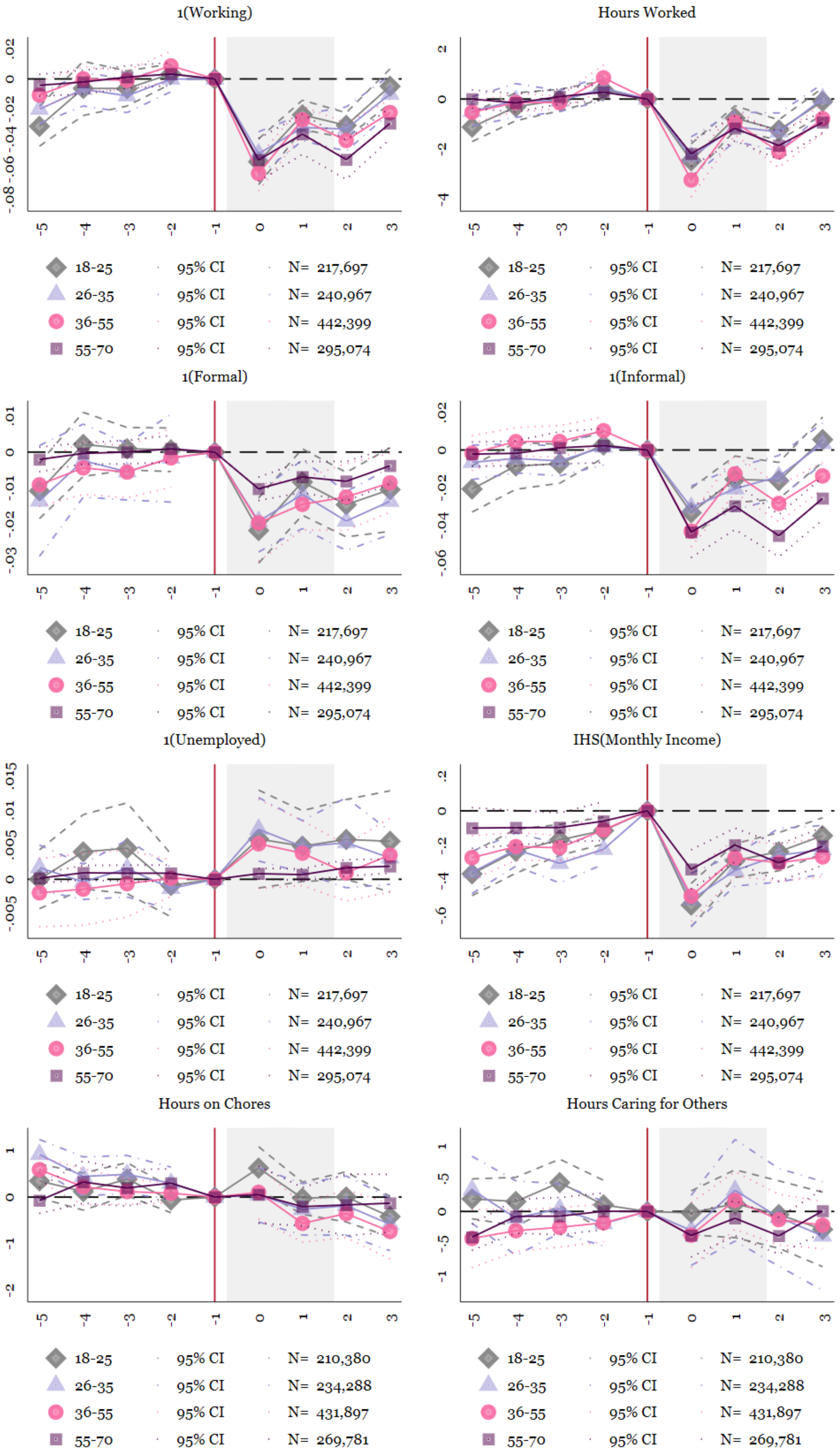

NOTES: OLS coefficients reported from Equation 1. Baseline fixed effects include state-level fixed effects. Controls include indicators for the individual's age and education. Sample weights are applied using the ENOE's specified sample weights. The periods before -1 include 2019Q1-2019Q4. The omitted period represents 2020Q1, represented by the vertical line. There is no 2020Q2, so the post periods represent 2020Q3-2021Q2. The shaded period represents quarters in 2020. The main sample includes individuals who are 18 to 64. Robust standard errors are clustered at the state level. Individual-level data from the traditional face-to-face National Occupation and Employment Survey (ENOE) 2019Q1-2020Q1 and 2020Q3-2021Q2.

Figure D.5: Event Study: By Age Group for Men

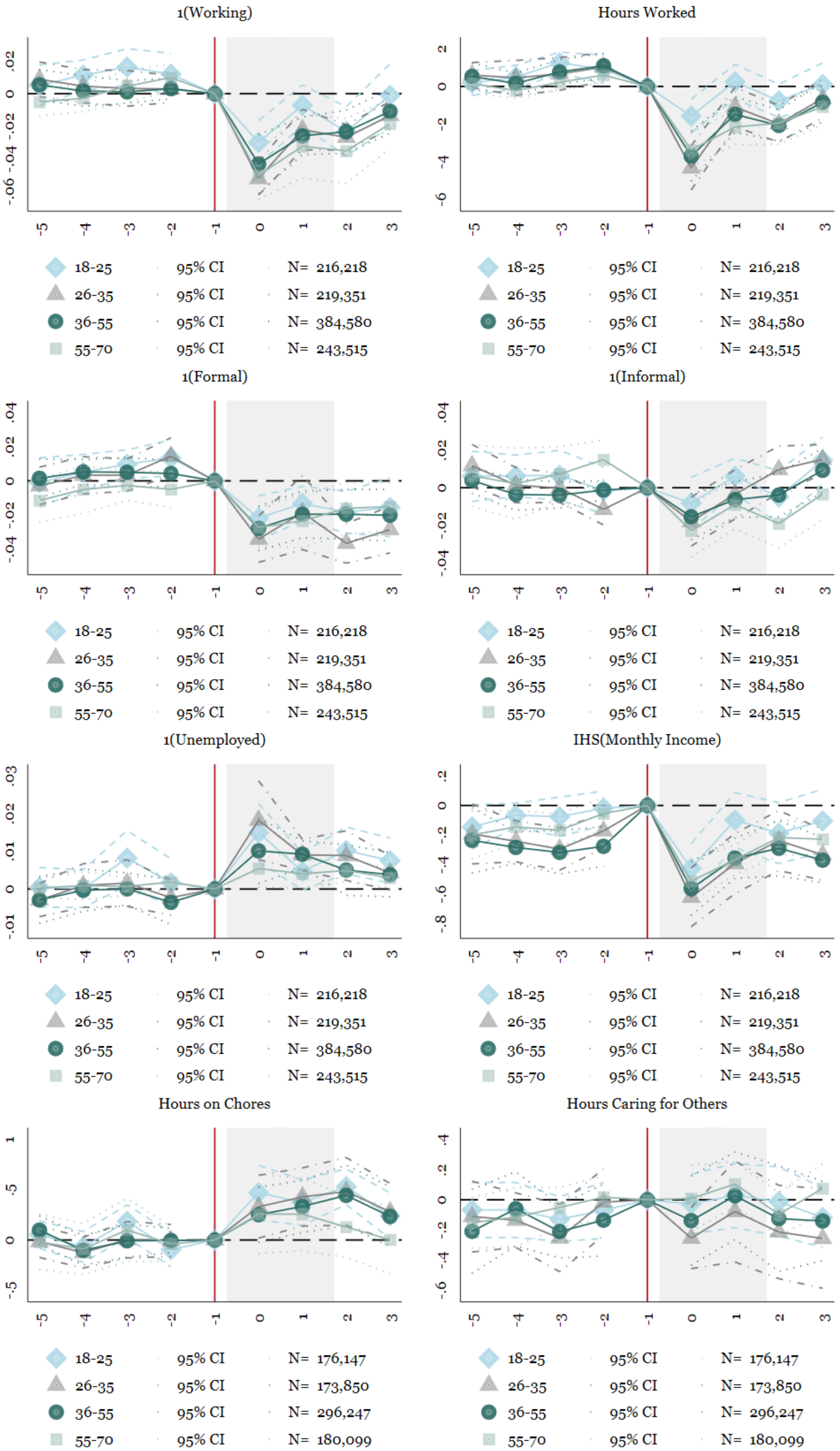

NOTES: OLS coefficients reported from Equation 1. Baseline fixed effects include state-level fixed effects. Controls include indicators for the individual's age and education. Sample weights are applied using the ENOE's specified sample weights. The periods before -1 include 2019Q1-2019Q4. The omitted period represents 2020Q1, represented by the vertical line. There is no 2020Q2, so the post periods represent 2020Q3-2021Q2. The shaded period represents quarters in 2020. The main sample includes individuals who are 18 to 64. Robust standard errors are clustered at the state level. Individual-level data from the traditional face-to-face National Occupation and Employment Survey (ENOE) 2019Q1-2020Q1 and 2020Q3-2021Q2.

Figure D.6: Event Study: By Education Group for Women

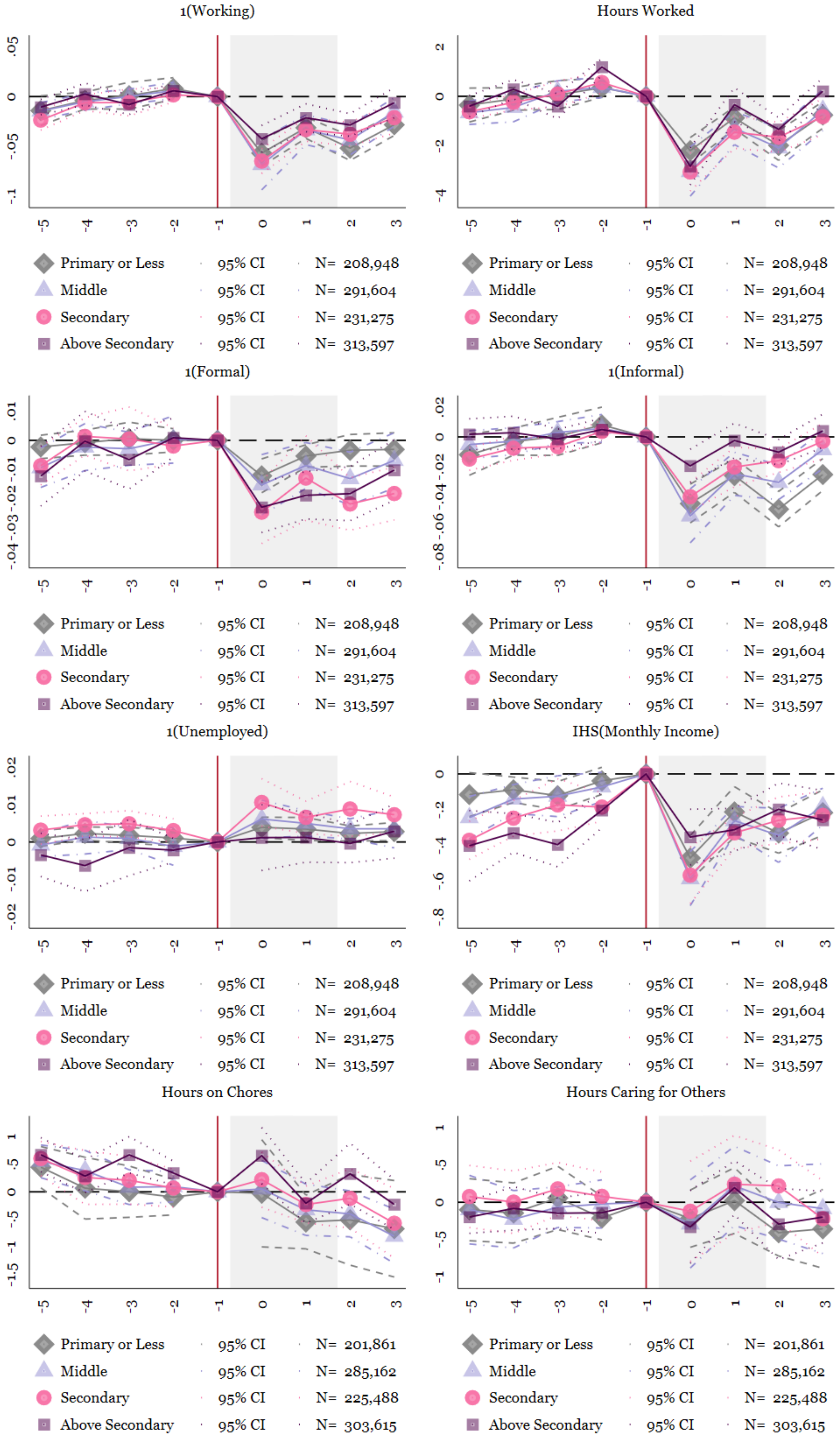

NOTES: OLS coefficients reported from Equation 1. Baseline fixed effects include state-level fixed effects. Controls include indicators for the individual's age and education. Sample weights are applied using the ENOE's specified sample weights. The periods before -1 include 2019Q1-2019Q4. The omitted period represents 2020Q1, represented by the vertical line. There is no 2020Q2, so the post periods represent 2020Q3-2021Q2. The shaded period represents quarters in 2020. The main sample includes individuals who are 18 to 64. Robust standard errors are clustered at the state level. Individual-level data from the traditional face-to-face National Occupation and Employment Survey (ENOE) 2019Q1-2020Q1 and 2020Q3-2021Q2.

Figure D.7: Event Study: By Education Group, Men

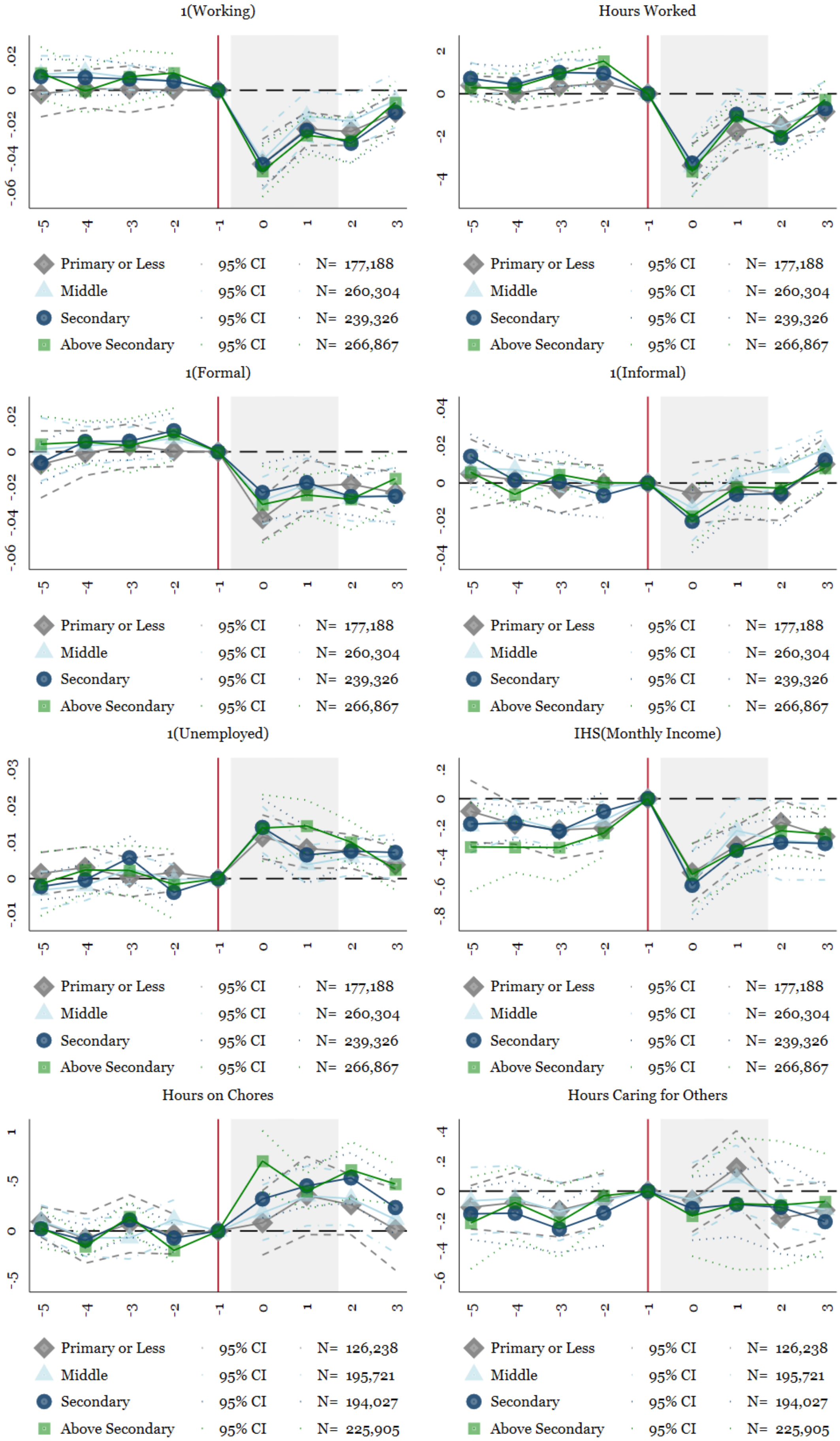

NOTES: OLS coefficients reported from Equation 1. Baseline fixed effects include state-level fixed effects. Controls include indicators for the individual's age and education. Sample weights are applied using the ENOE's specified sample weights. The periods before -1 include 2019Q1-2019Q4. The omitted period represents 2020Q1, represented by the vertical line. There is no 2020Q2, so the post periods represent 2020Q3-2021Q2. The shaded period represents quarters in 2020. The main sample includes individuals who are 18 to 64. Robust standard errors are clustered at the state level. Individual-level data from the traditional face-to-face National Occupation and Employment Survey (ENOE) 2019Q1-2020Q1 and 2020Q3-2021Q2.
